# Predicting procedural outcomes in transcatheter aortic valve implantation: a scoping review of numerical patient-specific simulations

**DOI:** 10.1088/2516-1091/ae1772

**Published:** 2025-11-06

**Authors:** Benedetta Grossi, Letizia Maria Perri, Valeria Raona, Ottavia Cozzi, Francesco Migliavacca, Gianluigi Condorelli, Giulio Stefanini, Giulia Luraghi

**Affiliations:** 1 IRCCS Humanitas Research Hospital, Via Alessandro Manzoni 56, 20089 Rozzano, Milan, Italy; 2 Department of Chemistry, Materials and Chemical Engineering, Politecnico di Milano, Piazza L. da Vinci 32, 20133 Milan, Italy; 3 Department of Biomedical Sciences, Humanitas University, Via Rita Levi Montalcini 4, 20072 Pieve Emanuele, Milan, Italy; 4 Fondazione IRCCS Cà Granda Ospedale Maggiore Policlinico, Milan, Italy

**Keywords:** TAVI, clinical outcome prediction, patient-specific numerical simulations, systematic review

## Abstract

Transcatheter aortic valve implantation (TAVI)-related post-operative complications remain significant clinical challenges, and current in-silico simulations fall short in predicting them accurately, limiting their clinical applicability. This scoping review evaluates the state of the art in TAVI computational modeling, identifying methodological gaps and proposing directions for refinement to enhance translational impact. Following PRISMA-ScR guidelines, 40 studies were included, with data extracted and summarized by evaluated outcomes. A quality assessment was performed using a 14-item rubric. Most studies focused on predicting paravalvular leak (65%) and conduction disturbances (20%). This review reveals substantial heterogeneity in modeling approaches, with limited standardization and varying degrees of validation. To improve clinical relevance, future efforts should prioritize model standardization, rigorous validation following ASME V&V guidelines, increased automation, and improved interpretability for clinical users. By ensuring robustness, efficiency, and clinical accessibility, in-silico models could transform TAVI outcome prediction and support personalized treatment planning, ultimately enhancing care standards in structural heart interventions.

## Abbreviations

TAVI: Transcatheter aortic valve implantation
PVL: Paravalvular leakage
THV: Transcatheter heart valve
FEA: Finite element analysis
CFD: Computational fluid dynamics
FSI: Fluid-structure interaction
LVOT: Left ventricular outflow tract
PPM: Permanent pacemaker
TAV: Transcatheter aortic valve
BCs: Boundary conditions

## 1 Introduction

Aortic stenosis is the most common valvular heart disease in developed countries. As a manifestation of aging, the condition is becoming more common as the average age of the population rises and severe symptomatic disease is fatal if left untreated. In this context, TAVI has emerged as a minimally invasive alternative to traditional surgical aortic valve replacement, particularly for high-risk patients [1].

While it has revolutionized the management of aortic stenosis, post-operative complications such as PVL, conduction disturbances, and coronary obstruction remain significant clinical challenges. Additionally, as the indication for TAVI expands to include lower-risk patients, there is an increased emphasis on the early diagnosis, proper management, and prevention of such complications [2]. This shift necessitates a more robust approach to addressing and minimizing complications for a broader patient population.

**Figure 1:**
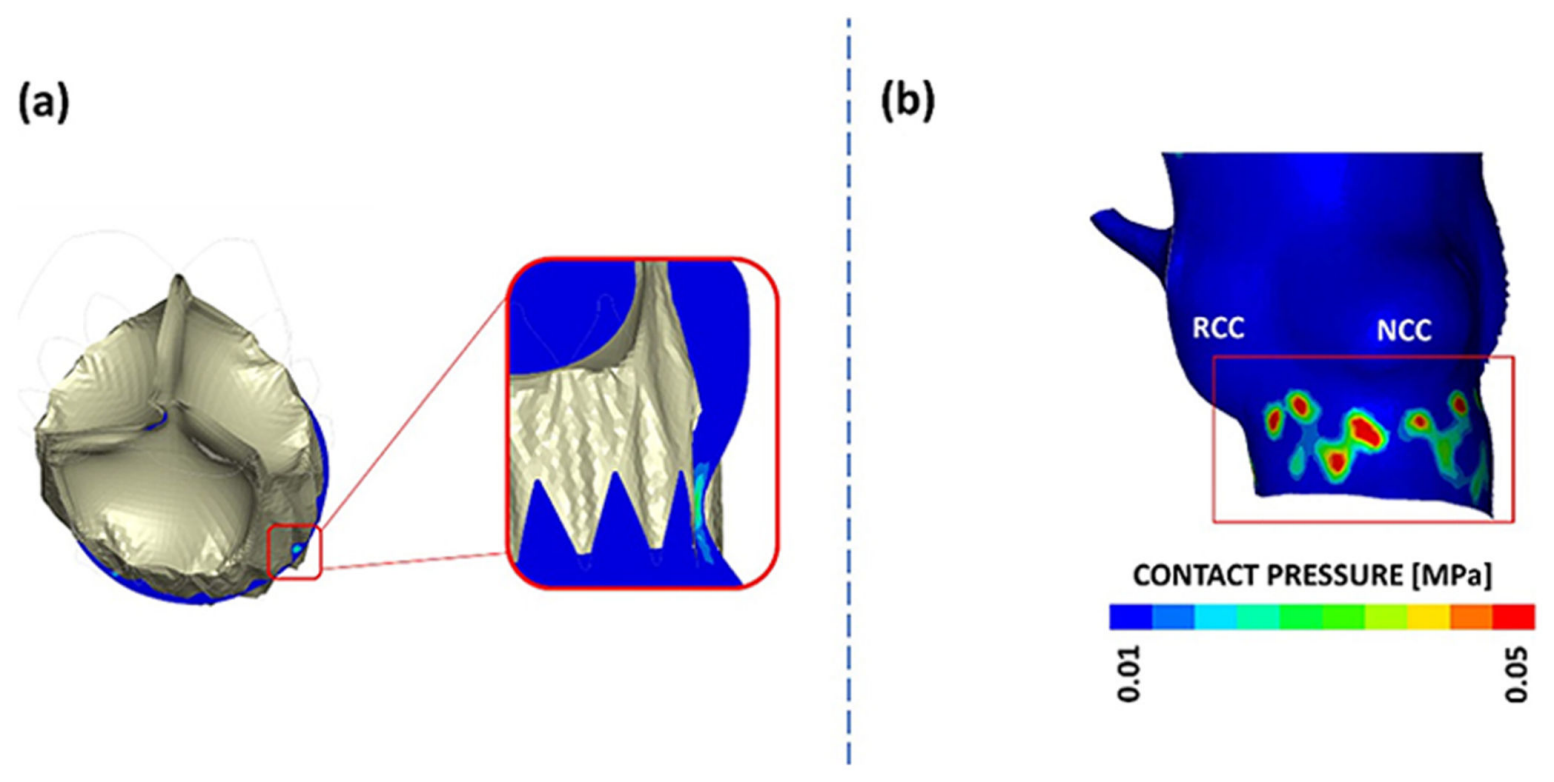
Post-TAVI outcomes prediction through in-silico simulation techniques. Legend: exemplary applications of computational modeling in predicting TAVI complications: (a) paravalvular leak assessment via fluid–structure interaction simulations; (b) contact pressure mapping to evaluate the risk of post-procedural conduction disturbances.

These statements highlight the potential crucial role of patient-specific simulations, which use computational modeling to replicate an individual’s anatomical and physiological characteristics and to predict TAVI complications (figure 1). By incorporating patient-specific anatomical and haemodynamic features, these simulations aim to enhance the accuracy of TAVI planning and execution, ultimately improving clinical outcomes. Specifically, several studies have employed methods such as FEA, CFD, and FSI models to replicate the TAVI procedure and evaluate its results. FEA is commonly used to investigate the mechanical response of the device, including stent deformation, radial force distribution, and interactions with the calcified native leaflets and surrounding aortic tissue. This approach provides valuable insights into device performance, procedural safety, and potential complications during critical procedural steps such as crimping, deployment, and anchoring. CFD models assess hemodynamic performance by examining blood flow patterns, pressure distributions, and complications such as PVL. FSI approaches combine FEA and CFD, enabling the study of interactions between the prosthetic valve and blood flow, thereby delivering a more comprehensive understanding of device performance under physiological conditions. Table 1 provides an overview of these three major modeling approaches in TAVR research, highlighting their respective strengths, clinical outcomes best suited for prediction, and commonly employed validation strategies.

However, despite advances in computational modeling, current in silico models still struggle to accurately predict these complications, limiting their practical application in real-world settings.

Several challenges contribute to these limitations. In particular, the variability of modeling assumptions and methodologies across the literature—such as differences in material properties, discretization methods, and computational techniques—leads to inconsistent or inaccurate outcome predictions. Additional factors further hinder reliability, including inconsistencies in patient-specific anatomical modeling techniques, insufficient validation against clinical data, and the omission of critical biomechanical aspects.

To address these shortcomings, this scoping review aims to comprehensively evaluate the state of the art in TAVI computational modeling. By analyzing existing studies, identifying methodological gaps, and highlighting inconsistencies, this review seeks to clarify where current models fall short and propose directions for refinement to enhance their translational potential in clinical practice.

## 2 Materials and methods

### 2.1 Review design

This scoping review was conducted in alignment with the guidelines outlined in the preferred reporting items for systematic reviews and meta-analyses extension for scoping reviews (PRISMA-ScRs) [3], as well as established methodological frameworks for scoping reviews [4]. In accordance with PRISMA-ScR, critical appraisal of the individual sources of evidence was considered optional.

### 2.2 Study selection

### 2.2.1 Literature sources, search strategies and selection process

The search process was conducted independently by three authors (B.G., L.M.P., V.R.) with expertise in bioengineering and medicine. This process involved performing the systematic search, selection of studies based on inclusion and exclusion criteria, data extraction, and management of the collected information. The systematic search was carried out on 10 January 2025, across three major databases: PubMed (MEDLINE), Scopus, and Web of Science. No filters were applied beyond limiting the results to articles from the last ten years and in the English language. The search strategy was designed using an approach similar to the Patient, Intervention, Comparison, Outcome framework. However, in this case, the search was structured around two main categories: computational simulation (e.g. virtual, simulation∗, FEA, computational simulation) and TAVI (e.g. TAVI, transcatheter aortic valve replacement, transcatheter aortic valve). These categories included multiple entry terms and Medical Subject Headings, ensuring the complete capture of relevant studies. To facilitate the retrieval of studies, within each category, the entry terms and Medical Subject Headings terms were combined using the OR operator, while the two main categories were connected using the AND operator. Once an effective search string was established for PubMed, it was adapted for use in both Scopus and Web of Science to ensure consistency across all three platforms.

To streamline the selection process, Rayyan software, a web-based automated screening tool developed by Qatar Computing Research Institute, was used to remove duplicates records and to do the screening of the studies based on abstract first, and full text, later. Furthermore, the reference lists of the included studies were examined to uncover any additional relevant studies that may not have been identified in the initial search. No automation tools were employed throughout this process. The study selection phase was completed on 24 February 2025.

#### 2.2.2 Inclusion and exclusion criteria

Original English-language articles replicating in-silico TAVI procedure to evaluate and predict clinical outcomes were selected. Exclusion criteria were: (1) articles different from original research papers (e.g. case reports, review articles, comments, and editorials); (2) background articles not referring to the prediction of a specific clinical outcome; (3) models not including a patient-specific anatomy and a realistic device reconstruction; (4) outcomes in terms of device performance only; (5) studies presenting non-numerical simulations; (6) analyses conducted on a patient cohort which differs from the intended population of patients with severe aortic stenosis (e.g. patients with aortic regurgitation).

### 2.3 Data gathering

Relevant data from the included studies were extracted and organized into pre-defined tables using Microsoft Word (Microsoft Corp, Redmond, WA). The tables were structured to facilitate easy comparison across studies. Information was initially gathered on study characteristics and evaluated outcomes.

#### 2.3.1 Study characteristics

Included papers were classified according to their main characteristics, including the first author, publication year, article type, study location, study aim, and any notable distinguishing features (e.g. simulation type and evaluated outcomes).

#### 2.3.2 Evaluated outcome and methodological details

Studies were also gathered on the evaluated outcome, highlighting methodological details. These included input data (e.g. patient population, device category) and numerical data (e.g. segmentation input, computational methods, aortic and THV models in terms of discretization and material properties, and other simulation components). Additionally, both qualitative and quantitative clinical, numerical, and comparative outcomes were recorded.

### 2.4 Quality assessment

The quality assessment was carried out independently by two authors (B.G., L.M.P.), who evaluated each study using a 16-item rating rubric [5]. To ensure consistency and accuracy, any discrepancies between the reviewers were resolved by a senior author (F.M.), who provided the final consensus where needed. Although the rubric was originally developed to assess research focused on simulations as a teaching methodology for physicians and nurses, it was adapted to 14 items for this review to better suit the numerical studies being evaluated. Among the 14 items used as evaluation criteria, particular attention was given to the robustness and completeness of the methodology, the qualitative and quantitative description of the simulation development, as well as the reporting of results and discussion. An important factor in the assessment was the number of patients included, and the variety of devices used in the study, as well as whether the study had received approval from an ethics committee.

The studies were then categorized by quality: a score below 50% indicated low quality, a score between 50% and 70% was considered intermediate quality, and a score above 70% was regarded as high quality.

### 2.5 Data presentation

Data were presented in textual format, with numerical values reported as number (n) and percentage (%).

## 3 Results

### 3.1 Study selection

**Figure 2:**
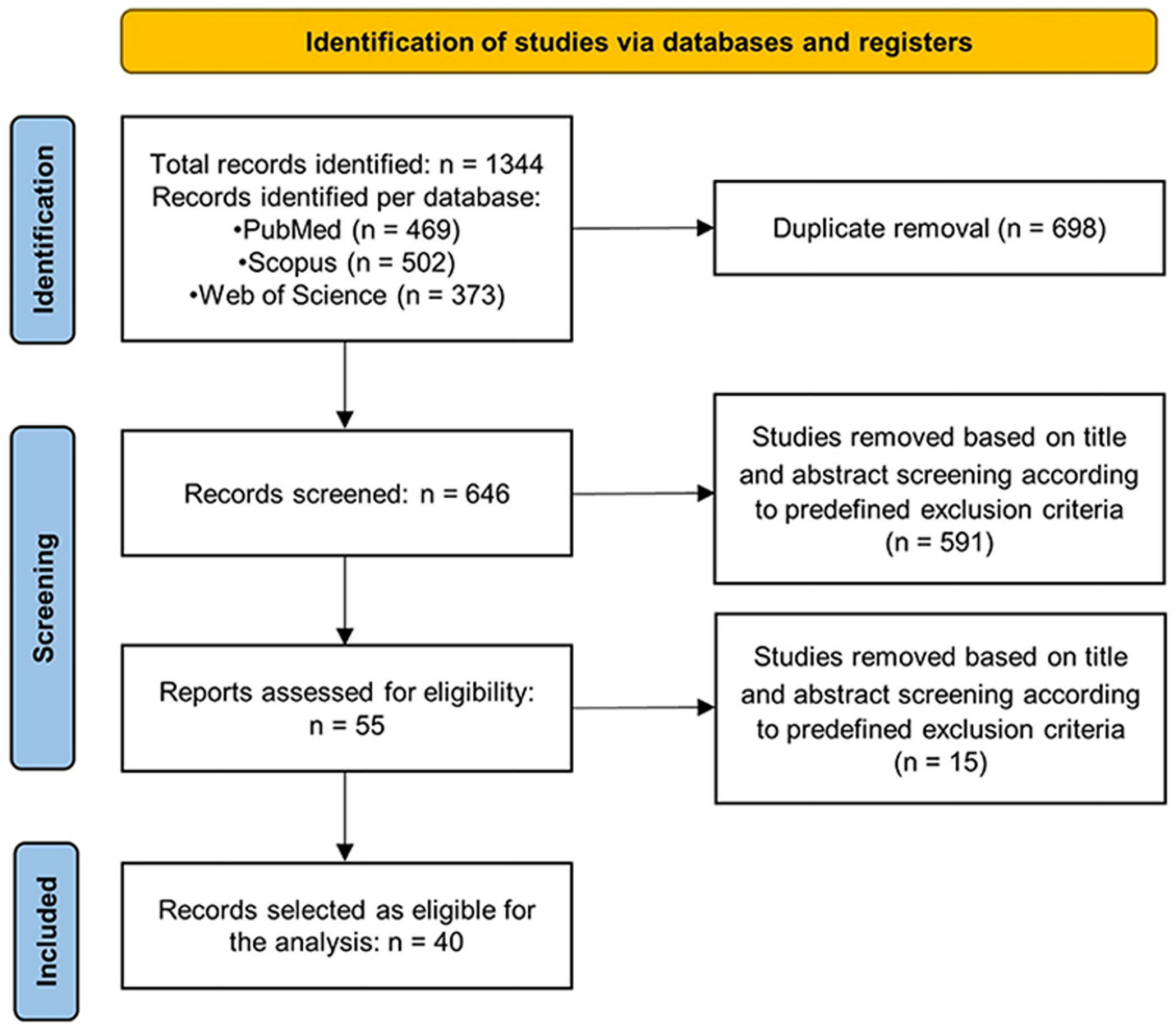
PRISMA-ScR flow diagram. Legend: PRISMA 2020 flow diagram for new systematic reviews used for identification, screening and inclusion of reviewed papers.

The initial search across three major databases identified a total of 1344 articles. After removing duplicates, 646 unique articles remained for title and abstract screening. Of these, 541 articles were excluded for not meeting the predefined eligibility criteria, leaving 55 articles for full-text review. Following a thorough assessment based on inclusion and exclusion criteria, 40 articles were selected for the final analysis [6–45]. The complete PRISMA-ScR flow diagram is presented in figure 2.

### 3.2 Study characteristics

The included studies are detailed in Table 2, organized in chronological order of publication. Among these, the majority—26 studies [6–9, 11–13, 16, 18, 20, 22, 24–29, 32, 34, 36–39, 41, 43] (65%)—were published in engineering journals. Additionally, 9 studies [14, 15, 17, 23, 30, 31, 35, 44, 45] (22.5%) were published as clinical works, and the remaining 5 studies [10, 19, 21, 33, 42] (12.5%) appeared in multidisciplinary journals.

When categorizing the studies according to their primary research focus, 26 studies [8, 11, 12, 15–17, 19–22, 24, 25, 27–33, 35, 39–43, 46] (65%) investigated PVL quantification as the main outcome. Meanwhile, 8 studies [15, 17, 20, 30, 34, 35, 39, 45] (20%) examined the impact of TAVI on conduction disturbances, assessing its association with EKG changes and pacemaker implantation risk. In addition, 3 studies [7, 13, 36] (7.5%) explored the risk of coronary obstruction, while 8 papers [14, 21, 23, 26, 27, 37, 38, 42] (20%) examined various aspects of thrombogenic risk, assessing how TAVI might contribute to clot formation and subsequent complications. Furthermore, 3 studies [7, 13, 36] (7.5%) investigated valve displacement, evaluating factors that contribute to improper valve positioning. Notably, only one study [9] specifically examined the relationship between TAVI-induced stresses and annulus rupture.

Regarding the computational methodologies employed to simulate TAVI outcomes, the studies utilized three primary approaches. FEA was the sole computational technique used in 14 studies [7–11, 14, 19, 20, 23, 25, 27, 34, 44, 45] (35%), primarily to model structural deformations and mechanical stress distributions. Meanwhile, 15 studies [12, 15–17, 21, 26, 30, 31, 33, 35, 39–43] (37.5%) incorporated CFD into their frameworks for the analysis of blood flow-related complications. Additionally, 12 studies [6, 13, 18, 21, 22, 24, 28, 29, 32, 36–38] (30%) leveraged FSI simulations, integrating both structural and hemodynamic aspects to achieve a more comprehensive representation of TAVI-related biomechanical behavior.

Among the different software used for these simulations, Abaqus (Dassault Systèmes Simulia Corp., Johnston, RI) emerged as the predominant choice for finite element modeling, with 84.2% of FEA-based studies relying on this tool for structural analysis. However, a greater diversity of computational tools was observed in blood flow simulations, reflecting the varied methodologies adopted for CFD and FSI studies.

### 3.3 Outcome evaluation methods

#### 3.3.1 PVL

**Figure 3:**
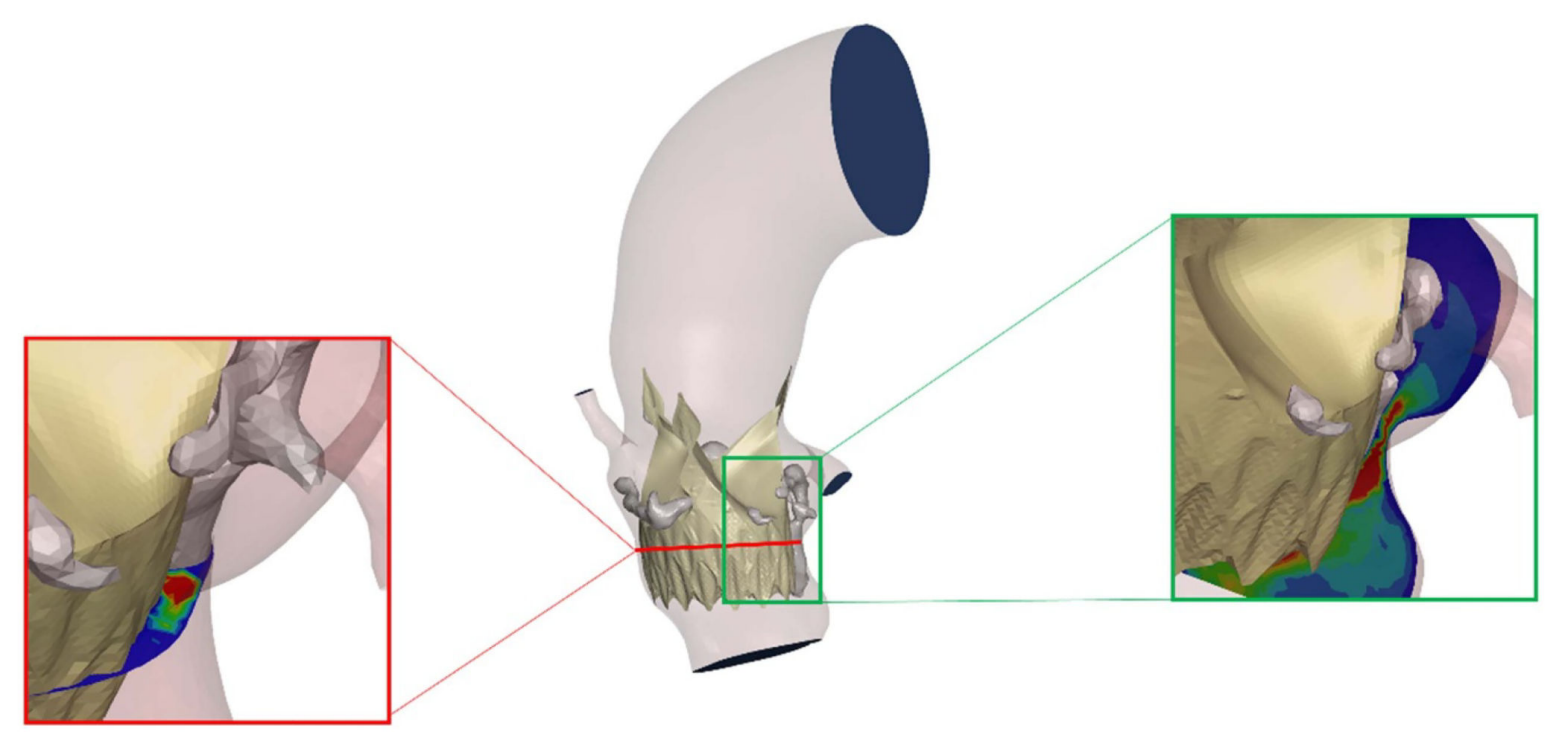
Exemplary CFD-based PVL evaluation. Legend: the model was able to replicate post-operative significant leak. The figure shows two cross-sectional planes that were placed midway through the leak to quantify flow across the defect.

Table 3 provides a comprehensive overview of the studies included in this review that investigated PVL. A total of 26 studies [8, 11, 12, 15–17, 19–22, 24, 25, 27–33, 35, 39–43, 46] (65%) met the inclusion criteria and focused on this clinical outcome. An illustrative example of PVL quantification is shown in figure 3.

Among them, 5 publications [8, 11, 19, 20, 25] (19.2%) relied solely on FEA methodologies to quantify PVL. Specifically, 3 studies [8, 11, 20] (7.5%) estimated leakage by measuring the distance gap between the aortic root and the simulated prosthetic device. Finotello *et al* [25] calculated the paravalvular orifice area by summing the total area of gaps between the inner aortic wall (including calcifications) and the outer stent surface. Zhang *et al* [19] developed a support vector regression model to examine the relationship between aortic stress and the risk of aortic regurgitation, identifying these stresses as the primary parameter of interest.

15 of the selected papers [12, 15–17, 21, 26, 30, 31, 33, 35, 39–42] (57.7%) utilized CFD methodologies to quantify regurgitant flow volume, providing a direct estimation of PVL severity by modeling the fixed diastolic scenario. Additionally, 7 studies [6, 21, 22, 24, 28, 29, 32] (26.9%) employed FSI simulations to model blood flow and assess PVL dynamics. In this context, Luraghi *et al* [6, 22] applied a non-boundary-fitted FSI method to evaluate regurgitant volume and the effective regurgitant orifice area. Pasta *et al* [24, 28, 29] used smoothed particle hydrodynamics in FSI simulations to compute mean particle velocity and characterize flow jets within the leakage gap. Lastly, Li *et al* [32] localized and quantified paravalvular gaps by analyzing the blank area between the stent and the aortic root at the annulus plane, utilizing FSI simulations based on the immersed boundary method.

#### 3.3.2 Conduction abnormalities

**Figure 4:**
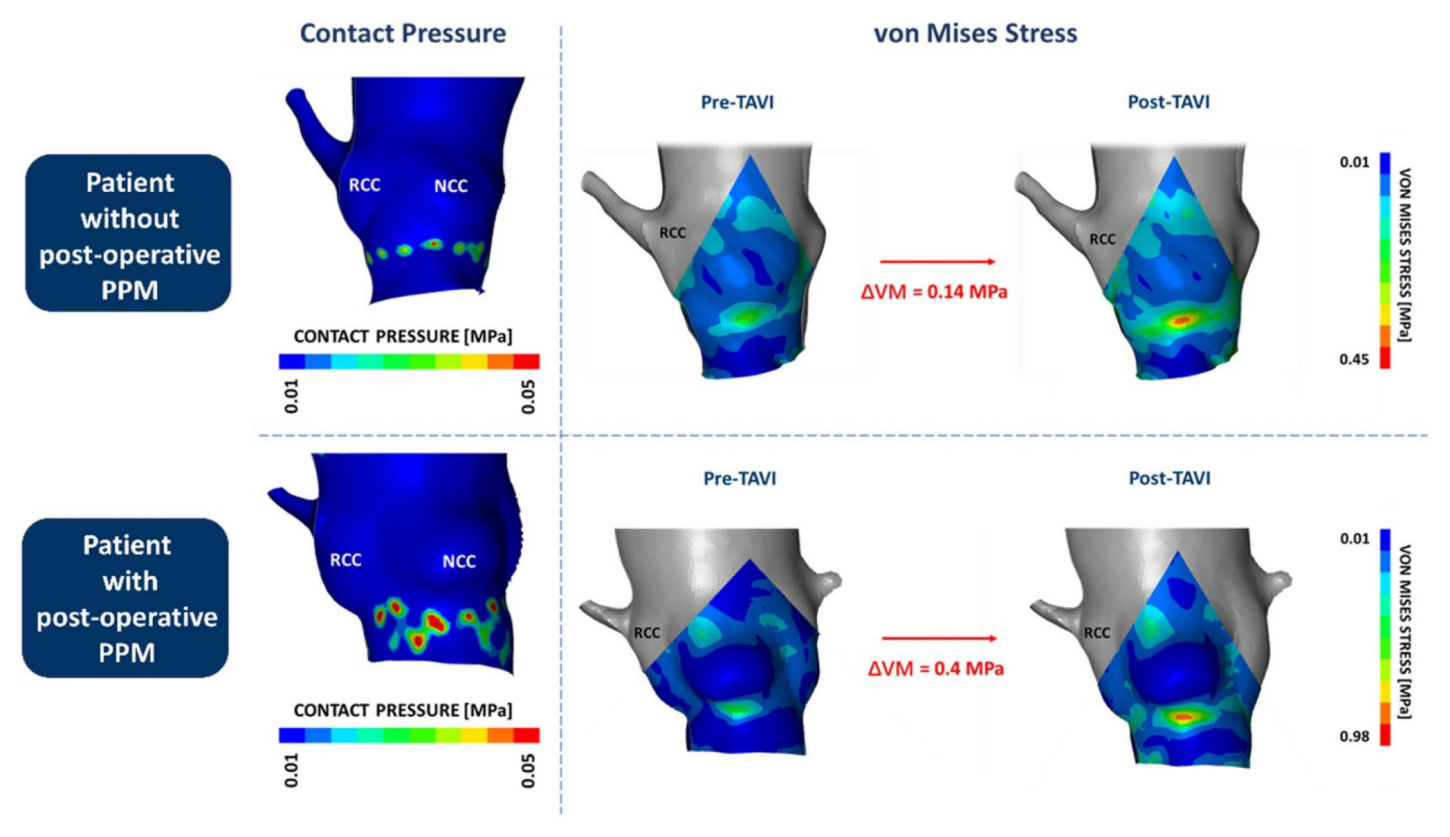
Exemplary FEA-based prediction of conduction disturbance risk after TAVI. Legend: representative FEA–derived maps of contact pressure and von Mises stress distribution before and after TAVI implantation. The upper panel shows a case without post-operative permanent pacemaker (PPM) implantation, characterized by peak contact pressures confined to the virtual basal ring and minimal extension into the LVOT. Correspondingly, the von Mises stress gradient between pre- and post-implantation remained low, indicating limited additional mechanical load on the aortic wall. In contrast, the lower panel illustrates a case requiring post-operative PPM, where a markedly higher von Mises stress gradient and broader peak contact pressure distribution extending across the LVOT suggest increased mechanical interaction between the stent frame and the conduction system.

Table 4 provides a comprehensive overview of the studies that investigate conduction abnormalities risk. Notably, 8 articles [15, 17, 20, 30, 34, 35, 39, 45] (20%) meeting our inclusion criteria focused on this critical outcome. These papers utilized FEA methodologies to predict the risk of conduction abnormalities development. An illustrative example of a study predicting post-TAVI conduction abnormalities is shown in figure 4.

The parameters analyzed included the maximum contact pressure within a defined region of interest—specifically, the region of the LVOT containing the atrioventricular conduction system—and the contact pressure index, which represents the percentage of the region of interest exposed to maximal pressure. Among these studies, 6 articles [15, 17, 30, 35, 39, 45] exclusively examined on these two parameters, while Reza *et al* [34] also considered the area-weighted average maximum principal logarithmic strain. Bosi *et al* [20], instead, focused solely on the maximum principal strains, which presented with higher values in patients requiring PPM implantation after the procedure. Patient-specific FEA has proven useful in identifying individuals at increased risk of post-TAVI conduction disturbances [35, 39, 44]. Rocatello *et al* [45] found that contact pressure and contact pressure index, rather than implantation depth, are associated with new conduction abnormalities. Dowling *et al* [15] established cutoff values for these parameters, while Rocatello *et al* [17] combined them to enhance prediction accuracy.

#### 3.3.3 Coronary obstruction

Out of the 40 selected articles, only 3 articles [7, 13, 36] (7.5%) investigated the risk of coronary obstruction, as summarized in Table 5. Capelli *et al* [7] employed FEA to analyze how different stent landing positions within the aortic root influenced coronary occlusion risk. The minimum distance between the leaflets and coronary ostia was studied. Kandail *et al* [13] combined FEA and FSI by simulating annular and supra-annular deployment, incorporating a finite-volume based sub-grid geometry method to refine flow calculations. Key parameters included instantaneous velocity and wall shear stress patterns. Oks *et al* [36] explored of the effects of three different commissural alignment angles on coronary perfusion using morphing functions and two-way immersed boundary FSI simulations. The investigated parameters are the mean systolic effective orifice area, the diastolic von Mises stresses and the coronary flow rate.

#### 3.3.4 Thrombogenic risk

Table 6 provides a detailed description of the studies included in this review that aim to assess the thrombogenic risk. Specifically, 8 articles [14, 21, 23, 26, 27, 37, 38, 42] (20%) that met our inclusion criteria focused on this critical outcome.

Nappi *et al* [14, 23, 27] (37.5%) utilized only FEA to quantify it. They evaluated the effects of stent distortion and malposition on late leaflet thrombosis, along with the calculation of von-Mises stresses to identify the areas associated with a major risk for aortic wall inflammatory changes. 3 of the selected papers utilized CFD methodologies [21, 26, 42] (37.5%). Papers belonging to this category mainly focused on the analysis of stress accumulation and on hemodynamic alterations (e.g. flux velocity, stasis volume and vorticity flux) that potentially may result in platelets activation. 3 articles [21, 37, 38] (37,5%) modeled blood flow with FSI simulations. In this context, Oks *et al* [37] used an immersed two-way FSI coupling method to evaluate the transvalvular pressure gradient, the geometric orifice area, stress accumulation and the wall shear stresses. Baylous *et al* [38] performed strong FSI simulations based on the Arbitrary Lagrangian–Eulerian approach, assessing the thrombogenic risk with Lagrangian particle tracking approach and stress accumulation analysis.

#### 3.3.5 Stent migration

3 of the 40 articles analyzed [10, 18, 21] (7.5%) aim to study stent migration after TAV deployment, as reported in Table 7. Among these 3, Bianchi *et al* and Gosh *et al* [10, 21] conducted FEA. The former calculates the contact area and pressure between the native leaflets and the stent during the deployment and recoil phases, while the others focus on the anchorage contact area and force between the stent frame and the native calcific aortic valve over time. Wu *et al* [18] built an immersed boundary FSI analysis, during which they computed radial and friction forces to assess the anchoring capability of the THV.

#### 3.3.6 Aortic root rupture

**Figure 5:**
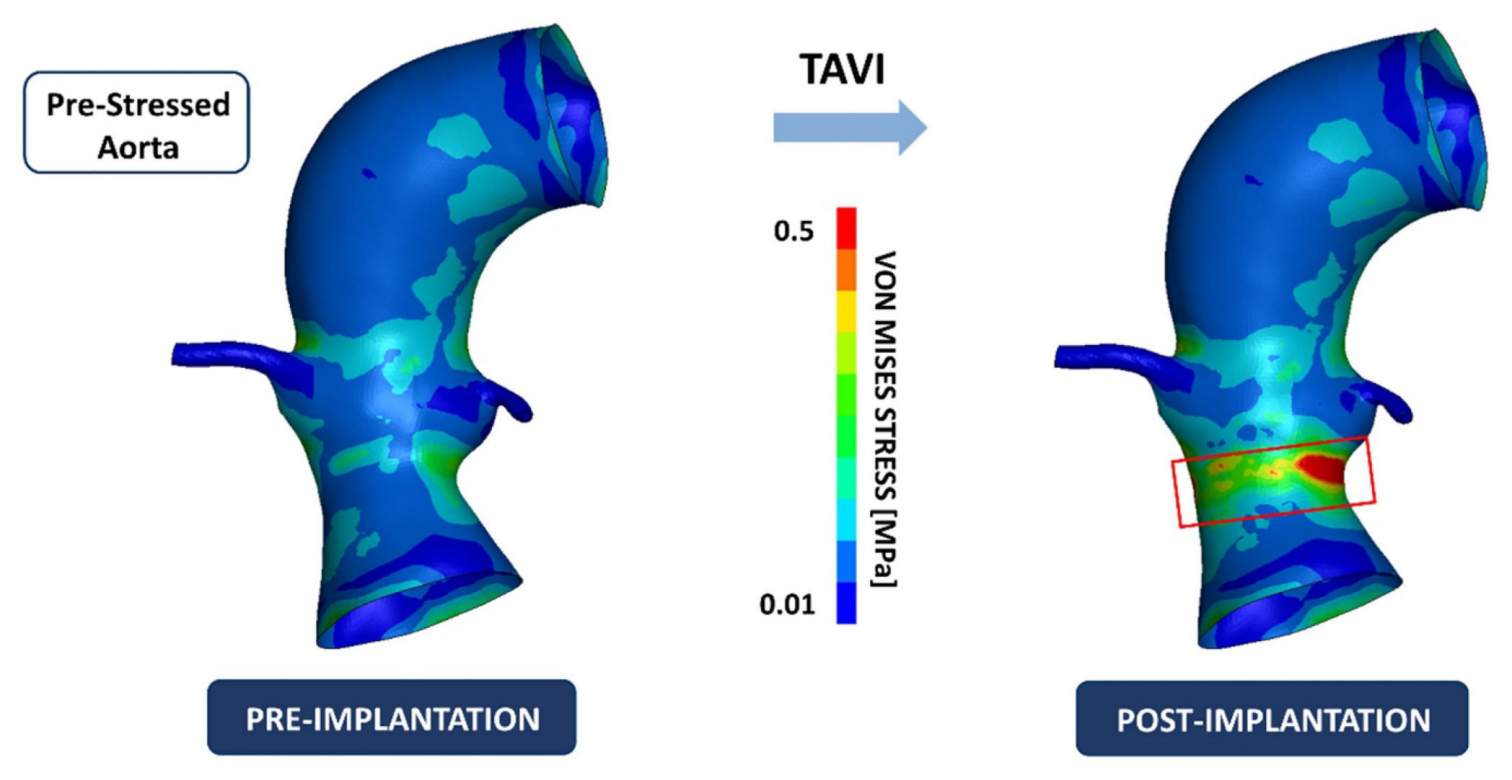
FEA-based simulation of post-TAVI aortic rupture risk. Legend: von Mises stress contour plots showing the stresses exerted by the implanted prosthetic valve on the aortic wall, used to assess the potential risk of rupture following TAVI.

A single study by Wang *et al* [9] investigated the risk of aortic rupture. Using FEA TAV deployment simulations, the contact force between the stent and the aortic root, as well as the deformed geometry of the aorta, were analyzed to evaluate rupture risk. Interestingly, the study found that pressure and force values were not correlated with aortic rupture, making crucial the study of patient-specific anatomical features and calcification patterns. An exemplary FEA-based prediction of post-TAVI aortic rupture is reported in figure 5.

### 3.4 Methodological details

#### 3.4.1 Anatomical model

Tables 3–8 provide an overview of the anatomical modeling details across the included research works. On average, each study analyzed 35.2 *±* 101.1 patients, with 11 papers [14, 15, 17, 19, 20, 23, 30, 31, 35, 44, 45] (27.5%) evaluating more than 10 cases. Among the studies, 12 [15, 16, 24, 25, 28–31, 35, 38, 42, 43] (30%) specifically focused on bicuspid aortic valves.

Most investigations (87.5%) derived patient-specific aortic anatomies from pre-operative computed tomography scans. 5 studies [18, 21, 22, 36, 40] (12.5%) have derived the geometry of the aortic arch either from already validated 3D models, parametric models, or by using average and statistical dimensions. On the other hand, none of the included studies performed direct reconstruction of native aortic valve leaflets from pre-operative imaging data.

Regarding the discretization of the anatomical domain, different approaches were adopted. The aortic root was represented using shell elements in 10 publications [7, 11, 17, 20, 24, 28, 29, 39, 40, 43] (25%), while solid elements (hexahedral or tetrahedral) were employed in 17 [6, 8–10, 12, 14, 16, 18, 19, 22, 25, 27, 32, 37, 38, 41, 42] (42.5%). The remaining 13 studies [13–15, 21, 23, 26, 30, 31, 33, 35, 36, 44, 45] (32.5%) did not specify the method used. Similarly, for the native valve, shell elements were applied in 12 cases (30%) [6, 11, 12, 20, 22, 24, 25, 28, 29, 39, 40, 42], whereas solid elements (hexahedral, prism, or tetrahedral) appeared in 10 [8–10, 14, 17, 21, 32, 34, 37, 43] (25%). Again, 18 works [7, 13, 15, 16, 18, 19, 23, 26, 27, 30, 31, 33, 35, 36, 38, 41, 44, 45] (45%) lacked details.

Regarding calcifications, solid elements were the preferred choice in 19 publications [6, 10, 11, 14, 16, 17, 19–22, 24, 25, 28, 29, 32, 34, 37, 39, 43] (47.5%), while 21 [7–9, 12, 13, 15, 18, 23, 26, 27, 30, 31, 33, 35, 36, 38, 40–42, 44, 45] (52.5%) omitted discretization specifics. Material modeling strategies also varied across the studies. The aortic root was described using a linear elastic model in 10 papers [15, 17, 20, 30–32, 35, 39, 44, 45] (25%), whereas 26 [6–12, 14, 16, 18, 19, 21–25, 27–29, 34, 37, 38, 40–43](65%) adopted a hyperelastic constitutive law. 1 study [33] (2,5%) did not specify the material properties. 3 of them characterized the aorta as a rigid material [13, 26, 37].

A similar trend was observed for native leaflets, with 14 studies [6, 12, 15, 17, 20, 23, 30, 31, 35, 37, 39, 42, 44, 45] (35%) implementing linear elastic models and 13 [10, 11, 14, 21, 22, 24, 25, 27–29, 34, 38, 43] (32,5%) using hyperelastic formulations, while 11 [7–9, 13, 16, 18, 19, 32, 33, 40, 41] (27,5%) provided no information. 2 articles [26, 36] (5%) described native leaflets as rigid bodies.

Calcifications were characterized as linear elastic in 20 cases [6, 8–10, 12, 16, 21–24, 27–29, 32, 34, 38, 39, 41–43] (50%) and elasto-plastic in 9 [15, 17, 20, 25, 30, 31, 35, 44, 45] (22.5%), whereas 10 studies [7, 11, 13, 18, 19, 26, 33, 36, 37, 40] (25%) omitted material property details. Nappi *et al* [14] described calcific plaques using hyperelastic properties.

#### 3.4.2 Device model

Device modeling details are summarized in tables 3–8. In 8 studies [7–10, 24, 26, 33, 34] (20%), patients only implanted balloon-expandable TAVs, specifically the Edwards Sapien, Sapien 3 and Sapien 3 ULTRA, and Sapien XT models, were included. In contrast, 22 papers [6, 11, 13, 16, 18, 21–23, 25, 28, 30, 31, 35–37, 39–45] (55%) focused solely on self-expandable devices, such as the Medtronic CoreValve, Evolut R, Evolut Pro, Evolut Pro+, Boston Scientific Acurate Neo2, the St. Jude Medical Portico valve, and Venus A-Valve. A combination of both self- and balloon-expandable devices was studied in 7 research works [12, 14, 20, 27, 29, 32, 38] (17.5%). Rocatello *et al* [17] employed the mechanically expandable Boston Scientific Lotus valve, while Dowling *et al* [15] examined both the Lotus and SAPIEN valves. Only one study by Zhang *et al* [19] did not specify the considered valve.

Regarding the virtual device reconstruction, a variety of approaches were employed. In 6 studies [6, 9, 13, 16, 32, 41] (15%), the model was reconstructed using a literature-based approach, relying on datasheets, technical specifications, and standards to derive the necessary dimensions and parameters. Alternatively, 12 papers [11, 14, 20, 24–29, 39, 40, 45] (30%) utilized physical device-based reconstruction, leveraging micro computed tomography scanning or optical microscopy to reverse-engineer the valve geometry. In 1 study [17] (2.5%), the geometric information was provided directly by the device manufacturer, while 2 works [10, 18] (5%) used parametric equations to estimate the expanded stent configuration. Dowling *et al* [15] utilized both micro computed tomography scanning, and data shared by device manufacturer. The reconstruction methodology was unspecified in 17 cases [7, 8, 12, 19, 21–23, 30, 31, 33–38, 42, 44] (42.5%).

Moreover, Anam *et al* [43] reconstructed geometric models using an in-house MATLAB code and ANSYS Spaceclaim. Additionally, only 18 studies [6, 10, 12, 13, 16, 18, 21, 22, 24, 26, 28, 29, 32, 36–38, 42, 43] (45%) included the pericardial TAV leaflets as part of the device model. Notably, Oks *et al* [37] only included the prosthetic leaflets in the FSI simulation. Spanjaards *et al* [40] included TAV leaflets in closed configuration.

Regarding discretization, various approaches were used across different components of the device. The stent frame was modeled using beam elements in 5 cases [11, 18, 20, 39, 40] (12.5%) and solid elements in 23 studies [6–10, 12–14, 16, 21, 22, 24, 25, 27–29, 32, 34, 37, 38, 41–43] (57.5%), specifically hexahedral in the majority of the cases [6–9, 12–14, 16, 21, 22, 24, 25, 28, 29, 32, 34, 37, 41–43] (50%). In 12 studies [15, 17, 19, 23, 26, 30, 31, 33, 35, 36, 44, 45] (30%), the discretization method for the prosthesis was not specified.

Among the 18 studies that include the prosthetic valve leaflets, shell elements were used in 12 papers [6, 10, 12, 16, 18, 22, 28, 32, 38, 40, 42, 43] (66.7%), either triangular or quadrilateral in shape. However, in 2 studies [21, 37] (11.1%) solid hexahedral, tetrahedral or pentahedral elements were employed for these components. Notably, Bianchi *et al* [10] did consider the leaflets but opted to not include them in the deployment models after a sensitivity analysis. 4 papers [13, 24, 29, 36] (22.2%) did not describe how the pericardium leaflets were discretised.

The material modeling of the metallic frame showed a high degree of consistency. The nickel-titanium alloy used for self-expanding devices was consistently modeled with a shape memory alloy formulation, while the cobalt-chromium frame of balloon-expandable devices was described using an elastoplastic material model. In terms of the prosthetic leaflets and skirt, material definitions varied more. These components were modeled as linear elastic in 8 papers [6, 13, 16, 22, 24, 29, 42, 43] (44.4%), hyperelastic in 8 studies [10, 18, 21, 28, 32, 36–38] (44.4%). Bianchi *et al* [12] considered the self-expandable TAV leaflets as linear elastic, while the balloon-expandable ones as hyperplastic. Spanjaards *et al* [40] and Hatoum *et al* [26] (11.1%) instead, considered skirt and TAV leaflets as rigid bodies in CFD simulations.

#### 3.4.3 Implantation simulation

FEA implantation simulations were conducted in 38 cases [6–25, 27–32, 34–45] (95%). Hatoum *et al* [26] and Prisco *et al* [33] extracted final implantation configuration directly segmenting post-operative computed tomography scans. None of these papers included physiological aortic pre-stress in the structural models.

**Figure 6:**
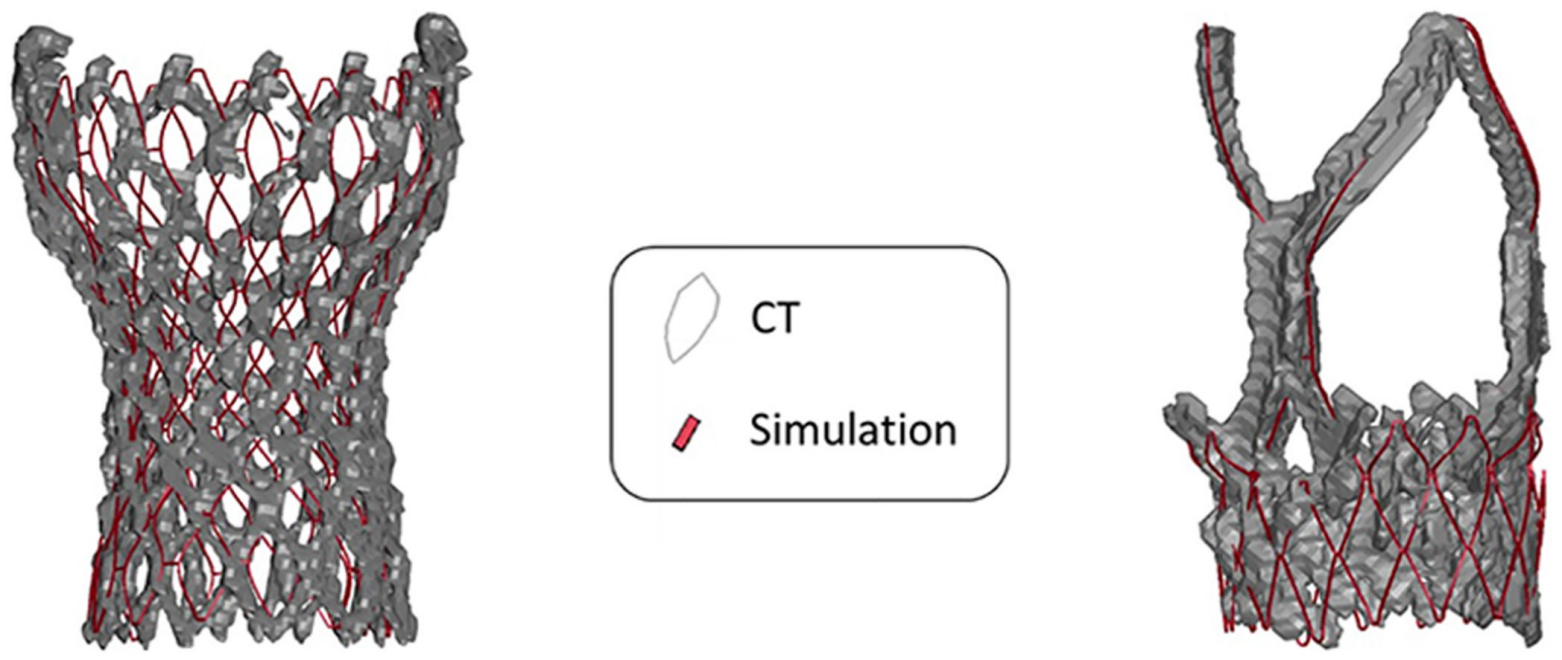
Validation of FEA implantation simulation using post-operative CT. Legend: the FEA simulation closely matches the post-procedural CT scan, as shown by the overlay, demonstrating the accuracy of the model.

Among the articles that considered the prosthetic leaflets, 11 papers [10, 12, 13, 21, 24, 29, 32, 37, 40, 42, 43] (61.11%) did not include the pericardial components during the deployment. Those studies carried out leaflets mapping on the deployed stent frame for the next analyses. 9 studies [11, 15, 20, 23, 24, 27, 30, 31, 35] (23.6%) mentioned validation of their finite element models using post-implant computed tomography images. An illustrative example of this validation process is shown in figure 6.

#### 3.4.4 Blood flow simulation

Among the selected studies, 26 [6, 12, 13, 15–18, 21, 22, 24, 26, 28–33, 35–43] (65%) performed blood flow simulations for predictive purposes. Within this group, the majority [6, 12, 15–17, 21, 22, 24, 28–33, 35, 39–43] (20 papers, 76.9%) focused on PVL quantification.

Regarding the simulation methods, CFD analysis was conducted in 14 studies [12, 15–17, 21, 26, 30, 31, 33, 35, 39, 41–43] (53.8% of blood flow simulations). On the other hand, a more advanced FSI approach was adopted in 12 publications [6, 13, 18, 21, 22, 24, 28, 29, 32, 36–38] (46.2%), incorporating the deformation of valve leaflets under hemodynamic loads.

Regarding boundary conditions (BCs), steady-state BCs were applied in 8 studies [15–17, 30, 31, 35, 39, 40] (30.8%), involving the application of a constant pressure gradient across the valve, representing diastolic or systolic phases. To better replicate physiological hemodynamics, 18 authors [6, 12, 13, 18, 21, 22, 24, 26, 28, 29, 32, 33, 36–38, 41–43] (69.2%) employed time-varying inflow and outflow conditions, accounting for the pulsatile nature of blood flow during the cardiac cycle.

Additionally, patient-specific BCs, derived from clinical data such as Doppler echocardiography or magnetic resonance imaging-based flow measurements, were utilized in 8 cases [6, 12, 22, 26, 33, 41–43] (30.8%), ensuring a personalized simulation framework that reflects individual anatomical and physiological variations. In contrast, 18 studies [13, 15–18, 21, 24, 28–32, 35–40] (69.2%) relied on literaturebased BCs obtained from population-average data, opting for a more standardized approach.

**Figure 7:**
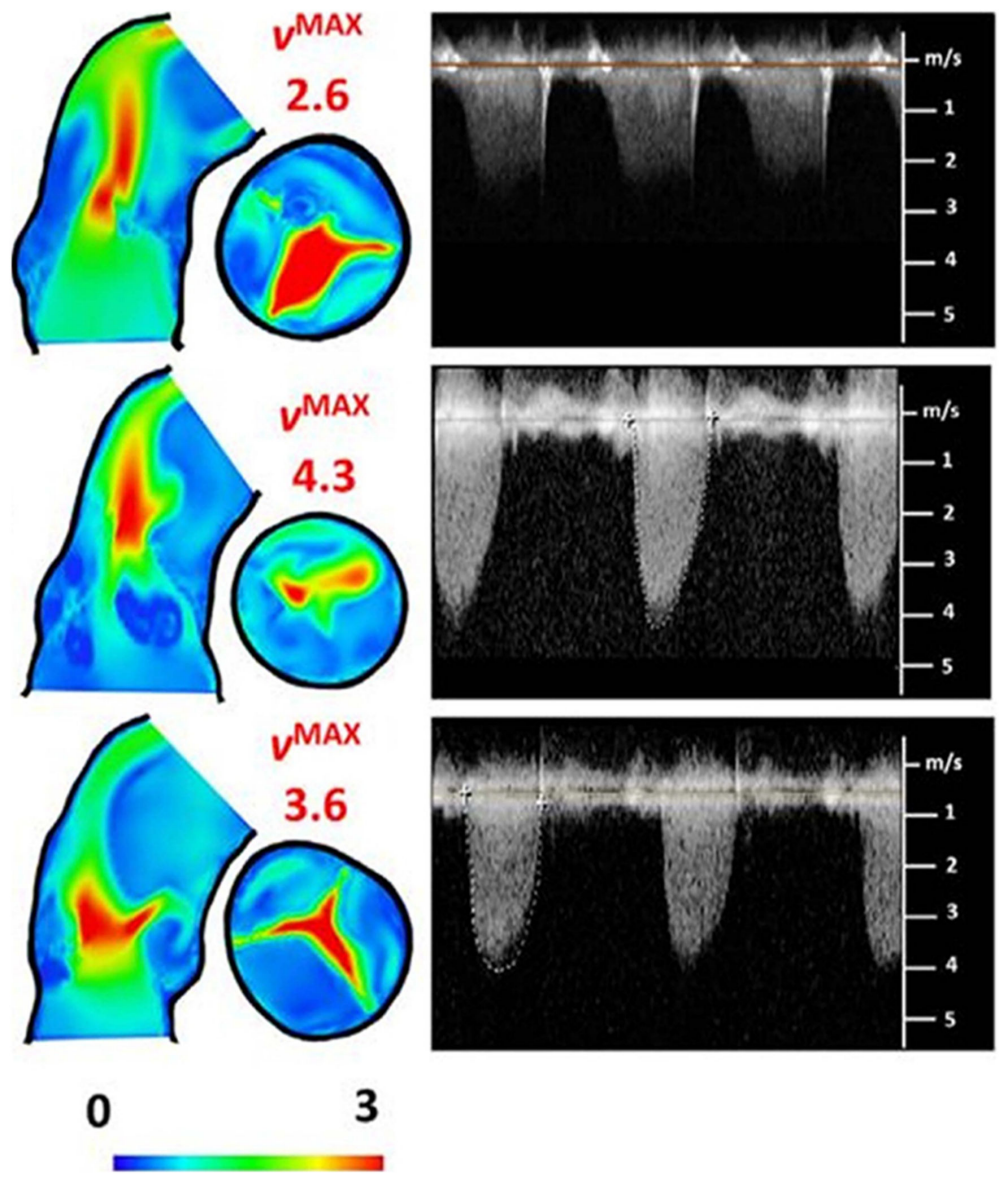
Validation of FSI simulations against post-operative clinical imaging data. Legend: left panels show the velocity field on longitudinal and transverse aortic sections for three representative patients, while the right panels display corresponding Doppler tracings for validation purpose.

Among these studies, 12 publications [6, 15, 17, 20, 24, 30, 31, 35, 38, 40, 42, 43] (46.1%), reported a validation process in which computational results were compared with post-operative clinical data, evaluating key hemodynamic parameters against echocardiographic or catheter-based measurements, or with experimental results. An example of validation using post-operative clinical imaging data is shown in figure 7.

### 3.5 Quality assessment

The quality assessment showed that 12 studies [8, 15, 19, 23, 27, 30, 32, 33, 35, 40, 41, 44] (30%) were of low quality, 19 studies [9, 11–14, 16, 18, 20, 24, 26, 28, 29, 31, 34, 39, 42, 43, 45, 47] (47.5%) of intermediate quality, and 9 studies [6, 7, 10, 17, 21, 22, 36–38] (22.5%) of high quality (Table 2).

## 4 Discussion

This scoping review aimed at comprehensively evaluating the state of the art in TAVI computational modeling, attempting to clarify where current models fall short and how they can be refined to be integrated in the clinical practice. To address this gap, we selected and analyzed TAVI modeling papers that specifically aimed at predicting patient-specific procedural complications, with a particular focus on methodological details.

The results from the reviewed literature highlight a significant degree of heterogeneity in TAVI modeling, underscoring the inconsistency in various computational approaches.

Regarding anatomical modeling, most studies derived patient-specific aortic anatomies from pre-operative computed tomography scans, which provided a consistent approach for capturing anatomical details. However, none of the included studies performed direct reconstruction of native aortic valve leaflets from pre-operative imaging data. This omission is likely due to the inherent challenges in segmenting the thin leaflet structures using standard computed tomography imaging, which often lacks the necessary resolution and contrast. Consequently, detailed patient-specific modeling of native aortic valve leaflets remains an area for future research and development.

The methods used to discretize the anatomical domain varied significantly across the studies. For instance, in the modeling of the aortic root, various studies have employed different finite element types. While some utilized shell elements to represent the geometry, others adopted solid elements, including both hexahedral and tetrahedral meshes. The choice between shell and solid elements often depends on the trade-off between computational cost and accuracy. While shell elements are computationally more efficient and may sufficiently capture the overall geometry of the aortic root, they can limit the accuracy of biomechanical assessments. In contrast, solid elements enable a more detailed representation of the tissue’s mechanical behavior, allowing for the simulation of complex deformations. This distinction is particularly relevant when predicting local stress and strain patterns, which are critical for evaluating device–tissue interactions during implantation procedures. Similarly, the native valve was often modeled using shell elements, as these are generally more efficient for modeling thin, flexible structures like leaflets. However, several studies adopted solid elements such as hexahedral, prism, or tetrahedral ones. When it came to calcifications, most studies opted for solid elements.

In terms of material modeling, a range of strategies was reported across the studies. Regarding the description of the aortic root, the linear elastic model, while simpler and less computationally expensive, may be insufficient for accurately capturing the non-linear and large deformations typically observed in soft biological tissues. In contrast, the hyperelastic model, which accounts for the large strains and non-linear material behavior of the aortic wall, is preferred in most studies, offering more realistic simulations. Also in this case, the decision to use a linear elastic model in some studies reflects a trade-off between model complexity and computational efficiency, but it might compromise the model’s ability to predict realistic behaviors in more complex scenarios. Despite these considerations, the aortic root was described using a linear elastic model in 30% of cases. A similar trend was observed for the native leaflets, where some studies employed linear elastic models, while others used hyperelastic formulations. The preference for hyperelastic material models likely stems from their capacity to more accurately represent the highly nonlinear and deformable behavior of the valvular tissue, particularly under the large strains experienced during device implantation and throughout the cardiac cycle. However, the use of linear elastic models can still be justified in scenarios where the primary focus is on capturing the global or macroscopic mechanical response of the leaflets, rather than detailed, localized tissue-level deformations. Additionally, the mechanical influence of the native leaflets may be considered negligible compared to that of the calcifications, particularly regarding their impact on device expansion. As a result, the specific modeling choices for the leaflets may have a less significant effect on the overall simulation outcomes than those related to the representation of calcifications.

For calcifications, most studies characterized them as linear elastic materials with a high Young’s modulus, which is appropriate, given the relatively rigid nature of these structures. However, some studies employed an elasto-plastic approach to account for the plastic deformations that may occur under high stress. This modeling choice is particularly relevant in pathological cases such as aortic stenosis, where calcifications lead to significant alterations in the mechanical properties of the valve. Further investigation in this direction could therefore be valuable to improve the accuracy of simulation-based predictions.

Device modeling was another major area of variability across the reviewed studies. The studies often focused on different types of prosthetic valves, including balloon-expandable and self-expandable TAVs. For instance, some studies only included patients implanted with balloon-expandable devices, such as the Edwards Sapien models, while others focused solely on self-expandable TAVs, such as the Medtronic CoreValve and Evolut models, and the Boston Scientific Acurate Neo2. A limited number of studies examined both types of devices, and a few others looked at mechanically expandable valves, such as the Boston Scientific Lotus valve. This wide variation in device types across studies indicates that the modeling approaches may differ based on the type of valve being used, which could affect the results and their applicability to different patient populations.

Regarding the virtual reconstruction of the devices, multiple strategies were employed. Some studies used a literature-based approach to derive the necessary dimensions and parameters, relying on datasheets, technical specifications, and standards. Other studies used physical device-based reconstruction techniques, such as micro computed tomography scanning or optical microscopy, which allow for more accurate reverse engineering of the device’s geometry. However, a few studies depended on information directly provided by the device manufacturers, and some used parametric equations to estimate the expanded stent configuration. These variations in reconstruction methods introduce variability in the device models, which can significantly influence the precision of the simulations. More accurate reconstructions, such as those based on physical device measurements or manufacturer-provided data, generally result in more reliable and detailed simulations. In contrast, models relying on approximations or generalized specifications may introduce errors, particularly affecting the accuracy of device–tissue interactions and stress distribution predictions.

Discretization of the device components varied across studies. The stent frame was typically modeled using either beam elements or solid hexahedral elements. However, it has been shown that beam elements can adequately capture the overall kinematics and final configuration of the stent, while remaining computationally more efficient [49]. For the pericardial leaflets and skirt, shell elements—usually triangular or quadrilateral—were commonly used. Some studies, however, opted for solid hexahedral or tetrahedral elements for these components.

Material modeling for the device components also showed substantial variability. The metallic frame, particularly in self-expanding devices, was consistently modeled using a shape memory alloy formulation for nitinol alloys, and the cobalt-chromium frame of balloon-expandable devices was modeled using an elastoplastic material law. However, for the valve leaflets and skirt, material models varied significantly. Many studies used linear elastic models, while others used hyperelastic or elastoplastic ones.

Additionally, several studies did not rigorously validate their models with experimental data. In fact, while some studies included validation of the material properties with experimental tests, the majority relied on literature-based material models. This lack of experimental validation raises concerns about the accuracy and applicability of the models for predicting real-world device behavior, especially when applied to patient-specific simulations.

In structural implantation simulations, several key biomechanical aspects are often overlooked. For example, none of the reviewed studies incorporated physiological aortic pre-stress in their implantation workflows. However, as demonstrated by Ramella *et al* [50], accounting for pre-stress is essential to accurately capture the real stress state of the aorta, including blood-induced deformations. Additionally, more than 60% of the studies did not consider the pericardial components of the device during deployment, instead opting for a post-procedural mapping of the leaflets and skirt. This approach can introduce errors, as it neglects the influence of the pericardial components on the stent behavior and fails to appropriately model the pericardium bending and the adaptation of valve leaflets during deployment. Furthermore, only 20% of the included studies mentioned validating their finite element models with post-implant computed tomography images, limiting the real-world applicability of these tools in clinical practice.

In blood flow simulations, more than 50% of the studies that modeled the fluid domain chose to use CFD simulations rather than an FSI approach. While CFD is a common choice, this strategy may be less accurate in TAVI modeling with respect to the FSI strategy, where the deformability of the structural components—particularly dynamic elements such as prosthetic leaflets or compliant aortic annulus—plays a critical role in the physical environment. Another key concern is the selection of BCs, which define the interaction between the model and the cardiovascular system. A large number of studies applied steady-state BCs, typically using a constant pressure gradient across the valve to represent the diastolic phase when quantifying PVL. However, time-varying inflow and outflow conditions more accurately reflect the pulsatile nature of blood flow, providing a more realistic representation of the cardiovascular cycle. Additionally, many studies did not incorporate patient-specific hemodynamics when defining their boundary conditions. As noted in the results section, only 30.8% of studies used patient-specific BCs derived from clinical data such as Doppler echocardiography or magnetic resonance imaging-based flow measurements. In contrast, the majority relied on literature-based BCs derived from population-average data, introducing potential inaccuracies in assessing patient-specific hemodynamics. Furthermore, only 30.8% of the papers included a rigorous validation process, comparing computational results with post-operative clinical data, such as echocardiographic or catheter-based measurements, or experimental results. The remaining studies did not report any validation, once again limiting the applicability of these models to real-world clinical practice.

A critical insight from this analysis is the widespread reliance on multiple semi-manual steps to obtain simulation results. These labor-intensive procedures—combined with the substantial computational time required—significantly extend the overall duration of the simulation workflow. Moreover, they necessitate the involvement of technical experts, which limits scalability and hinders broader integration into routine clinical practice. As a result, the application of these computational models remains largely confined to complex, high-risk cases where their added value justifies the additional time and resources.

Another key issue that emerged from this review is the lack of consistency in target variables used to define clinical outcomes. For instance, in assessing PVL, 5 studies [8, 11, 19, 20, 47] relied exclusively on FEA simulations, measuring parameters such as the gap distance between the aortic root and the implanted prosthetic valve. In contrast, studies utilizing blood flow simulations adopted different fluid dynamics indices to quantify regurgitant flow volume. This variability makes it difficult for clinicians to identify a single, standardized parameter that provides a clear interpretation of simulation results. As a result, these powerful computational tools often face scepticism and are not easily integrated into clinical decision-making.

Another challenge is the limited interpretability of biomechanical parameters for a clinical audience. For instance, most studies evaluating post-procedural conduction disturbances quantified contact pressure, a metric that holds little direct clinical relevance. As a result, the limited integration of in-silico medicine into clinical practice is, in part, due to the suboptimal translation of simulation outcomes into terms that are meaningful for clinicians. The gap between computational findings and real-world application complicates communication with clinicians, reinforcing the perception that these tools are far removed from practical use. However, as highlighted in this review, numerical simulations have demonstrated their ability to predict a wide range of outcomes, positioning them as potentially extremely powerful tools in current clinical practice. Notably, this work underscores a growing interest in the use of simulations to anticipate PVL and conduction disturbances, which remain among the leading contributors to suboptimal outcomes following TAVI procedure [51].

In conclusion, current models sometimes fall short in predicting clinical outcomes partially due to variations in modeling strategies, which can significantly affect the consistency and accuracy of the virtual replica. The lack of standardization across methodologies leads to discrepancies in predictive performance, particularly when assessing critical clinical endpoints. Moreover, these inconsistencies highlight the challenge of creating universally applicable models that can reliably translate to clinical practice, where precision and reproducibility are paramount. The future direction of in-silico modeling could go forward a rigorous standardization of the modeling process, including a strong and robust validation in accordance with the Verification and Validation guidelines outlined by American Society of Mechanical Engineers. A representative example of this approach within the context of TAVI simulations is provided by a recent study from our group [52], where the reliability of the implantation modeling was assessed through both qualitative and quantitative comparisons with post-operative clinical data, including angiographic and computed tomography imaging. Such efforts are essential to increase clinical users’ confidence in these tools and to facilitate their integration into clinical decision-making, ultimately enhancing the quality of patient care. Additionally, a greater emphasis should be placed on enhancing the automation of the simulation process and improving the interpretability of biomechanical parameters to facilitate their adoption by clinical users. Translating numerical outputs into clinically accessible and actionable metrics is crucial for bridging the gap between engineering insights and medical application.

The implementation of these strategies could elevate in-silico simulations from exploratory tools to essential components of clinical decision-making. By ensuring reliability, robustness, automatization, and clinical interpretability, these models have the potential to revolutionize TAVI outcome prediction and enable truly personalized treatment planning, ultimately setting a new benchmark for patient care in structural heart interventions.

## Figures and Tables

**Table 1:** Comparative summary of computational modeling approaches applied in TAVR research. The table highlights the principal strengths of finite element analysis, computational fluid dynamics, and fluid–structure interaction, along with the procedure-related outcomes each method is best suited to predict and the most commonly employed validation strategies.

	Primary strengths	Predictable TAVR outcomes	Validation strategies
Finite element analysis (FEA)	Provides an accurate description of mechanical response, device-tissue interaction, and stress/strain distribution	Conduction disturbances, stent migration, annular rupture, coronary obstruction (geometry-based)	CT-based reconstructions, angiographic imaging, *in vitro* bench testing
Computational fluid dynamics (CFD)	Enables detailed quantification of blood flow, pressure fields, and shear stresses; allows visualization of flow patterns and jets; relatively rapid simulations compared to FSI	Paravalvular leakage, thrombogenic risk, coronary flow alterations	Doppler echocardiography, 4D flow MRI, *in vitro* bench testing
Fluid–structure interaction (FSI)	Couples structural mechanics and hemodynamics, enabling physiologically realistic, time-dependent simulations of device-flow interaction; provides the most comprehensive framework for assessing both mechanical and hemodynamic outcomes	Conduction disturbances, stent migration, annular rupture, paravalvular leakage, thrombogenic risk, coronary flow alterations	Doppler echocardiography, 4D flow MRI, *in vitro* bench testing

**Table 2:** Study characteristics of the 40 articles analyzed, including computational fluid dynamic (CFD), finite element analysis (FEA) and fluid structure interaction (FSI) simulations of the TAVI procedure, aim of the study and quality assessment. [PVL = paravalvular leakage, CT = computed tomography, TAV = transcatheter aortic valve, BAV = bicuspid aortic valve, THV = transcatheter heart valve; TAVI = transcatheter aortic valve implantation].

Author, year	Place	Journal focus	Simulation type	Aim of the study	Software	Evaluated Outcome	Quality assessment score
Capelli *et al* [7]	UK	Engineering	FEA	Investigation of the feasibility of TAVI in specific patient morphologies which are currently borderline cases for a percutaneous approach	Abaqus	Coronary obstruction	70
Wang *et al* [8]	USA	Engineering	FEA	Study of the biomechanical interaction between the stenotic aortic valve and TAV stent	Abaqus	PVL	48
Wang *et al* [9]	USA	Engineering	FEA	A better understanding of the biomechanical interaction between the tissue and stent for patients with a high risk of aortic rupture.	Abaqus	Annulus rupture	57
Bianchi *et al* [10]	USA	Multi-disciplinary	FEA	Evaluation of the effect of various TAVI deployment locations on the procedural outcome by assessing the risk for valve migration	Abaqus	Stent Migration	70
Bosmans *et al* [11]	Belgium	Engineering	FEA	Development and validation of a simulation workflow based on preoperative clinically available data, which allows to predict the post-operative geometry and estimate the paravalvular aortic regurgitation	Abaqus	PVL	55
Bianchi *et al* [12]	USA	Engineering	FEA + CFD	Development of a methodology to assess the effect of TAV implantation depth and balloon overinflation on post-procedural complications and to help in reducing their impact based on patient-specific data	Abaqus, Ansys Fluent	PVL	68
Kandail *et al* [13]	USA	Engineering	FEA + FSI	FSI simulations for a 29 mm CoreValve deployed in annular vs supra-annular locations, to characterize resulting hemodynamic including velocity and wall shear stress	Abaqus, FlowVision	Coronary obstruction	59
Mao *et al* [41]	USA	Engineering	FEA + CFD	Development of computational models to perform a parametric investigation of the impact of various TAVI shape, deployment and modeling strategies on PVL	Abaqus, Star-CCM+	PVL	50
Nappi *et al* [14]	**France**	Clinical	FEA	To study the mechanism of thrombus formation and device dislodgement in the presence of persistent calcific blocks with varying calcium score indices in series of prohibitive-high-risk patients who underwent catheter—based aortic valves intervention.	Abaqus	Thrombogenic Risk	54
Rocatello *et al* [45]	Belgium	Clinical	FEA	To investigate to what extent mechanical pressure, assessed by patient-specific computer simulations, affects the conduction system after TAVI	Abaqus	Conduction abnormalities	68
Dowling *et al* [15]	Australia	Clinical	FEA + CFD	To validate a patient-specific computer simulation of TAVI in BAV by comparing the output of computer simulations to postprocedural CT imaging, cineangiography, echocardiography, and electrocardiograms	Abaqus, OpenFoam	PVL and conduction abnormalities	48
Lavon *et al* [16]	Israel	Engineering	FEA + CFD	Examination of the influence of different orientations deployment of TAVI. CFD simulations to evaluate PVL severity and to compare Evolut R and Evolut PRO self-expandable valves	Abaqus, FlowVision	PVL	58
Luraghi *et al* [46]	Italy	Engineering	FEA + FSI	Development of a patient-specific FSI methodology able to model the implantation phase as well as the valve working conditions during the cardiac cycle	LS-Dyna	PVL	80
Rocatello *et al* [17]	Belgium	Clinical	FEA + CFD	Verification of the predictive power of computational modeling of postoperative aortic regurgitation and conduction abnormalities. Evaluation of the impact of device size and position in patients with equivocal aortic root dimensions	Abaqus, OpenFoam	PVL and conduction abnormalities	70
Wu *et al* [18]	USA	Engineering	FEA + FSI	Development a computational FSI framework for TAVI simulations to study THV anchoring and estimate the possibility of migration	Not specified	Stent migration	63
Zhang *et al* [19]	USA	Multi-disciplinary	FEA	Combination of bio-mechanical and machine learning modeling to create a pre-procedural planning tool to support clinical decision making reducing the medical cost and the risk related to mortality and unfavorable outcome of TAVI	ANSYS	PVL	36
Bosi *et al* [20]	Italy	Engineering	FEA	Testing of a validated patient-specific computational framework for prediction of TAVI outcomes and possible complications	Abaqus	PVL and conduction abnormalities	54
Ghosh *et al* [21]	USA	Multi-disciplinary	FEA + CFD + FSI	Assessment of TAVI valve deployment during heartbeat to obtain optimal implantation depth via FEA analysis, to compare post-deployment TAVI thrombogenic potential for different implantation depths using CFD simulations, and to calculate deployed TAVI valve PVL for different implantation depths using FSI simulations	Abaqus, ANSYS Fluent	PVL, stent migration and thrombogenic risk	75
Nappi *et al* [23]	France	Clinical	FEA	Development of a predictive model to evaluate the progression of thrombotic process with the aim to discuss current evidence for the use of this operation	Abaqus	Thrombogenic risk	45
Pasta *et al* [24]	Italy	Engineering	FEA + FSI	Development of a patient-specific computational framework to virtually simulate TAVI in stenotic BAV patients using the Edwards SAPIEN 3 and SAPIEN 3 Ultra TAVs, and quantify stent frame deformity as well as the severity of PVL	Abaqus	PVL	57
Dowling *et al* [31]	Australia	Clinical	FEA + CFD	Investigation of the role that patient-specific computer simulation might play in optimizing transcatheter heart valve sizing and positioning in BAV and of the use of computer simulation to assess the differences in predicted paravalvular regurgitation between several different bicuspid sizing and positioning strategies	Abaqus, OpenFoam	PVL	52
Finotello *et al* [47]	Italy	Engineering	FEA	Comparison of the performance of High Conformability against High Radial Forces stents in highly elliptic and calcified BAV patients	Abaqus	PVL	59
Hatoum *et al* [20]	USA	Engineering	CFD	Development of a new method to stratify the risk of leaflet thrombosis based on the valve geometric, anatomical, and flow related (hemodynamic) parameters	Ansys	Thrombogenic risk	66
Luraghi *et al* [22]	Italy	Engineering	FSI	Study of the impact of different calcific deposits pattern on the virtual procedure outcome	LS-Dyna	PVL	77
Nappi *et al* [20]	Italy	Engineering	FEA	Investigation of failed TAVI cases in order to identify elements or abnormalities potentially responsible for valve failure and to generate predictive complications’ models	Abaqus	PVL and thrombogenic risk	43
Pasta *et al* [28]	Italy	Engineering	FEA + FSI	Determination of the biomechanical implication of TAVI in severe stenotic bicuspid aortic valve by developing a computational framework to assess the region at risk of PVL	Abaqus	PVL	55
Pasta *et al* [29]	Italy	Engineering	FEA + FSI	Determination of the structural mechanics and hemodynamic of Evolut PRO and SAPIEN 3 THV	Abaqus	PVL	52
Anam *et al* [42]	USA	Engineering	FEA + CFD	Analysis of the PVL complications using CFD and flow study, investigating the device deformation and comparing the in silico and *in vitro* results	Abaqus, ANSYS Fluent	PVL	64
Anam *et al* [43]	USA	Multi-disciplinary	FEA + CFD	Demonstration of the potential of computational techniques to analyze post-TAVI PVL complications in patient-specific BAV models, assess the risk of PVL induced thrombogenicity and compare self-expandable device performances in the same patient anatomies	Abaqus, Ansys Fluent	PVL and thrombogenic risk	57
Dowling *et al* [30]	Australia	Clinical	FEA + CFD	Validation of the predictive power of CFD simulations and examination of the role that patient-specific computer simulation might play in identify patients at risk for long-term adverse outcomes after TAVI	Abaqus, OpenFoam	PVL	45
Dowling *et al* [44]	Australia	Clinical	FEA	Validation of the conduction disturbance modeling in patients treated with current-generation self-expanding THVs based on the hypothesis that patient-specific computer simulation could predict the development of conduction disturbance. Examination whether computer simulation could identify patients at risk for prolonged hospitalization and long-term adverse clinical outcomes on the hypothesis that the patient-specific computer simulations would also be predictive of these clinical outcomes.	Abaqus	Conduction abnormalities	43
Li *et al* [32]	China	Engineering	FSI	Evaluation of the difference of implantation outcomes between the balloon-expandable valve and the self-expandable valve	Abaqus, Ls-Dyna	PVL	46
Prisco *et al* [33]	USA	Multi-disciplinary	CFD	Demonstration that CFD modeling can predict the location and accurately quantify the PVL and to assess the relative contribution of the native valve in preventing PVL using an iterative approach	Autodesk CFD	PVL	43
Reza *et al* [34]	USA	Engineering	FEA	Employment of an advanced computational techniques to simulate balloon-expandable TAVI procedure in patient-specific anatomy and analyzed several different anatomical and mechanical factors to identify the best cardiac conduction abnormalities risk assessing parameter	Abaqus	Conduction abnormalities	59
Dowling *et al* [35]	Australia, USA	Clinical	FEA + CFD	Description of authors’ ongoing experience with patient-specific computer simulation of TAVI in BAV, and to discuss its potential role in Heart Team decision making within the bicuspid patient cohort	Abaqus, OpenFoam	PVL and conduction abnormalities	41
Oks *et al* [36]	Spain	Engineering	FEA + FSI	Study of the effects of three different commissural alignment angles on coronary perfusion	Alya	Coronary obstruction	73
Oks *et al* [37]	Spain	Engineering	FEA + FSI	Investigate the effect of the sinotubular junction diameter on the structural implantation, hemodynamic performance, and thrombogenic risk in TAVI patients	Abaqus, Alya	Thrombogenic Risk	77
Baylous, 2024	USA	Engineering	FEA + FSI	Assessment of TAVI device performance and thrombogenic risk using an advanced FSI framework	Abaqus, LS-DYNA	Thrombogenic risk	79
Meng *et al* [39]	China	Engineering	FEA + CFD	Development of numerical simulation methods for rapidly predicting post-TAVI outcomes and potential complications, and to compare the postoperative results of self-expanding valves at different implantation positions, contrasting them with clinical outcomes	Abaqus	PVL and conduction abnormalities	63
Spanjaards *et al* [40]	The Netherlands	Engineering	FEA + efficient leakage model + CFD	Implementation and validation of device deployment model combined with an efficient, simplified leakage model based on the thin-film approximation to calculate the regurgitant volume and assess the risk of PVL	RADIOSS, Ansys	Fluent PVL	50

**Table 3:** Methodological aspect of the PVL estimation, focusing on the model discretization, boundary conditions, validation details, and conclusions of the articles [CFD = computational fluid dynamics, FE = finite element, PVL = paravalvular leakage, MSCT = multi slice computed tomography, SE = self-expandable, BE = balloon-expandable, CT = computed tomography, BC = boundary conditions, LVOT = left ventricular outflow tract, TAV = transcatheter aortic valve, BAV = bicuspid aortic valve, THV = transcatheter heart valve, TAVI = transcatheter aortic valve implantation].

Author	Input data	Device model	Model discretization	Material models	Simulation steps and boundary conditions	Model validation	Analyzed parameter	Conclusion
Wang *et al* [11]	1 pre-operative MSCT with severe calcification	Balloon-expandable TAV (SAPIEN). TAV leaflets were not included, stent geometry design was not specified	Aortic root, leaflets and myocardium: hexahedral and tetrahedral elements Stent: hexahedral elements Balloon: shell elements	Aortic tissues: hyperelastic Stent: elastoplastic Calcifications: linear elastic	FE TAV deployment simulations	The model was neither clinically nor experimentally validated	Gaps between the aortic annulus and the stent	The potential of PVL can be evaluated from simulated post-deployment aortic root geometries
Bosmans *et al* [11]	10 pre-operative CTs	Self-expandable TAVs (Corevalve) with pre-dilatation. The stent was reconstructed from micro-CT scans, TAV leaflets were not included	Aortic root and native leaflets: triangular shell elements Calcium: linear tetrahedral elements Stent: quadratic beam elements	Aorta, native leaflets, and soft tissues: isotropic hyperelastic (Neo-Hookean) Stent: nitinol alloy	FE TAV deployment simulations Validation of the model with post-operative CT scans Prediction of the aortic regurgitation through a max-flow algorithm	The FE model was validated with post-operative CT scans. The percentage error between post-operative CT measurements and the in-silico results did not exceed 5%	Distance between the sealing skirt of the simulated device and the surrounding aortic root	The estimation of leakage based on the distance between the sealing skirt of the simulated device and the surrounding aortic root, showed promise, especially in the detection of very good implantations
Bianchi *et al* [12]	3 pre-implantation CTs	Balloon- expandable TAVs (SAPIEN) and self-expandable TAV (Corevalve). TAVs leaflets were included in the CFD simulations. Stent geometry design was not specified	TAV stent: hexahedral solid elements TAV leaflets: quadrilateral shell elements CoreValve cuff: quadrilateral shell elements SAPIEN cuff: triangular shell elements Aortic root: tetrahedral solid element Native valve: quadrilateral shell elements Balloon: quadrilateral shell elements	Aortic root: hyperelastic Native leaflets and calcific plaque: linear elastic Stent (SE): nitinol alloy Stent (BE): elasto-plastic SE leaflets and cuff: linear elastic BE leaflets: hyperplast ic (3rd order Ogden) BE cuff: linear elastic Balloon: linear elastic Blood: two-phase Newtonian fluid	FE TAV deployment simulations Fluid domain extraction and TAV leaflets mapping CFD simulations and PVL degree assessment PVL validation with post-deployment echo data. Aortic BCs: scaled pressure gradient waveform (dynamic BCs) to account for the increase in left ventricular pressure due to the aortic regurgitation Coronary arteries BCs: velocity waveforms	The model was neither clinically nor experimentally validated	Regurgitant volume	A computational approach by employing FE and CFD techniques to investigate post-TAVI hemodynamic in retrospective clinical cases affected by PVL was developed. The effect of implantation depth and balloon inflation volume on post-procedural complications provided useful insights on how the physician’s choice would impact the procedural outcome. Positioning was shown to influence PVL up to 47% in specific cases, thus leading to remarkably different post-procedural outcomes
Mao *et al* [41]	1 pre-operative MSCT images	Self-expandable TAV (CoreValve). The geometry was reconstructed from illustrations in literature, TAV leaflets were not included	Aortic root: 3D solid elements TAV stent: hexahedral elements	Human tissues: modified anisotropic hyperelastic (Holzapfel Gasser-Ogden material model) Mitral-aortic intervalvular fibrosa and fibrous trigones: isotropic hyperelastic (Ogden) Calcifications: linear elastic Stent: nitinol Blood: incompressible Newtonian fluid	Non-linear FE simulations of TAV deployment analyzing different implant orientations and heights, modeling the impact of three different skirt shapes on PVL CFD simulations using the post-TAVI geometries from the FEA BCs: Physiological pressure waveforms (dynamic BCs) applied to the ascending aorta and LVOT as the pressure inlet and outlet boundary conditions	The model was neither clinically nor experimentally validated	PVL flowrate, leaking flow velocity profile, volume rendering velocity fields, regurgitant volume, and cross section area of the leakage	The analysis of the effects of skirt shape, TAV orientation and deployment height on PVL provided useful insights into the deployment strategies for individual patient and may facilitate next-generation TAV designs. Because of the scallop shape of the skirt, the difference of PVL due to TAV orientation can be as large as 40%. This study also demonstrated that a rigorously developed patient-specific computational model could potentially serve as a tool to assist in pre-operative planning for TAVI deployment strategies to minimize PVL
Dowling *et al* [15]	37 pre-implant CTs of BAV patients	Self-expandable TAVs (5 CoreValve, 16 EvolutR, 2 Evolut PRO) and mechanical-expandable TAVs (14 Lotus). Stents’ morphology was derived from micro-CT scanning Strut width was obtained from optical microscopy or based on data shared by the device manufacturer. TAV leaflets were ignored	Not specified	Aortic tissue: elastic Native Leaflets: linear elastic Calcifications: elastoplastic TAV: not specified Blood: not specified	FE TAV deployment simulations, including pre- and post-dilatation, ignoring valve prosthetic leaflets and cuff CFD simulations to record the resulting flow Statistical analysis BCs: Fixed pressure gradient (static BCs) of 32 mmHg, derived from a population sample	The study validated patient-specific computer simulations by comparing predicted PVL with andpost-operative echocardiography. The results showed good accuracy of the model	Resulting flow	The authors have established that computer simulations may predict the development of more than mild PVR
Lavon *et al* [16]	1 pre-implant CT of a severely calcified BAV female patient	Self-expandable TAVs (Evolut R and Evolut PRO). Geometric model of the latest versions of the FDA approved devices were used. TAV leaflets were included	Aortic tissue and calcifications: tetrahedral elements Stent: hexahedral elements TAV leaflets: shell elements Cuff: membrane elements	Calcifications: linear elastic Aorta: hyperelastic (Odgen 3rd order) Stent: nitinol alloy TAV leaflets and cuff: linear elastic Blood: Newtonian fluid, slightly compressible	FE TAV deployment simulations for both types of valves Creation of five Evolut PRO models with different leaflet orientations CFD simulations of Evolut PRO to identify the orientation yielding the lowest PVL values CFD simulations of Evolut R to compare the results with Evolut PRO BCs: constant pressure of 90 and 0 mmHg (static BCs) employed in the aortic and left ventricle boundaries (average pressure gradient during the diastolic phase)	The model was neither clinically nor experimentally validated	Pressure and velocity contours, Volume flow	This study showed that the orientation and position of the inner cuff influence directly on the size of gap between the device and the calcified cusps, and as a result, on the amount of leakage through this gap. The optimal position occurs when the bioprosthesis commissures were aligned with the gap between the device and the fused cusp (and with the native commissures). Evoult R resulted with almost twice regurgitant flow volume compared to the Evolut PRO
Luraghi *et al* [46]	2 pre-implantation CTs of patients with severe aortic stenosis	Self-expandable TAV (CoreValve Evolut R). A parametrical CAD model of the device was created from literature data. TAV leaflets were included	TAV stent: hexahedral solid elements TAVskirt: triangular membrane elements TAV leaflets: quadrilateral shell elements Aorta: hexahedral solid elements Native valve: quadrilateral shell elements Calcifications: tetrahedral solid elements	Aortic root: anisotropic hyperelastic Native leaflets and calcific plaque: linear elastic Stent: nitinol alloy Pericardium: linear elastic Blood: incompressible Newtonian fluid	FE TAV deployment simulations including TAV leaflets Non-boundary fitted method FSI simulations. The operator split Lagrangian-Eulerian approach was used Calculated PVL validation with post-procedural clinical data. BCs: patient-specific pressure waveforms (dynamic BCs)	Qualitative validation of the FE model was performed against post-implant CT scans and angiographies, whereas the FSI velocity results were assessed against post-procedural Doppler traces. The comparison between the post-operative CT scan and the numerical model showed good qualitative agreement in terms of positioning. The estimated maximum velocity values were in agreement with the Doppler measurements	Regurgitant volume, effective regurgitant orifice area	The potentiality of the proposed methodology to reproduce two real clinical outcomes in terms of PVL was shown. This kind of numerical methodology could result very useful to guide clinical decision making beforeand after the procedure
Rocatello *et al* [17]	62 pre-operative MSCTs of patients with severe aortic stenosis	Mechanically expandable Lotus devices. Accurate device models of all Lotus valve sizes were generated based on information provided by the device manufacturer. Device leaflets were not included	Aortic root: triangular elements Native valve and calcifications: prism elements	Aortic tissue: elastic Native Leaflets: linear elastic Calcifications: elastoplastic Blood: incompressible fluid	FE TAV deployment simulations CFD simulations to quantify the blood flow during diastole to predict AR Predicted AR comparison to postoperative AR based on echocardiography and angiography Statistical analysis BCs: fixed pressure difference of 32 mmHg applied over the valve (static BCs)	In-silico results were compared to post-operative clinical data based on echocardiography and angiography. The results obtained showed good accuracy of the model	Aortic regurgitation flow	This study showed that patient-specific computational modeling and simulation can accurately predict postoperative aortic regurgitation
Zhang *et al* [19]	22 pre-procedural CTs	Not specified	Aortic roots and calcifications: hexahedral elements Other components: not specified	Aortic tissues: homogeneous isotropic hyperelastic Other components: not specified	FE TAV deployment simulations Support vector regression method model the relationship between the stress information and the risk of aortic regurgitation	The model was neither clinically nor experimentally validated	Distribution of stresses induced by the prosthetic valve	This study approach performed well in predicting aortic regurgitation for the aortic stenosis patients. The combination of biomechanical properties and machine learning method substantially improved prediction of clinical results.
Bosi *et al* [20]	28 pre-implant CTs	Ballon-expandable TAVs(14SapenXT) and self-expandable TAVs (14 CoreValve Revalving System). TAVI stent models were designed in their expanded configuration starting from micro-CT scans. Leaflets were not included	Stent (BE and SE): beam elements Balloon: membrane elements Aortic root and native leaflets: shell elements Calcium: tetrahedral elements	Stent (BE): elastoplastic Stent (SE): nitinol Aortic root and native leaflets: linear elastic Calcifications: elastoplastic Balloon: homogeneous isotropic linear elastic	FE TAV deployment simulations after pre-dilatation simulations Identification of potential PVL through an algorithm designed in house to quantify the gaps between artery and device	The FE model employed in this study had been previously validated [48]	Gap between implantation site and stent	The FE framework captured well the TAVI stent diameter at the end of the implantation procedure and the presence/lack of PVL. Considering the entire patient cohort, the mathematical model was able to identify the presence/lack of PVL in 83% of cases, thus demonstrating good sensitivity
Ghosh *et al* [21]	Avalidated 3D dynamic model of an adult beating heart that includes physiologically realistic structural and electrophysiological properties (Simulia Living Heart Human Model)	Self-expandable TAV (Evolut R). TAV leaflets were included. Stent geometry design was not specified	TAV stent and TAV leaflets: hexahedral solid elements TAV cuff: combination of hexahedral and tetrahedral solid elements Native valve and calcifications: tetrahedral solid elements	Heart tissue: Holzapfel-Ogden anisotropic hyperelastic material model Native leaflets and pericardium: Ogden isotropic hyperelastic material model Calcific plaque: linear elastic TAV Stent: nitinol alloy Blood: Newtonian fluid	FE TAV implantation simulation at three different implantation depths, ignoring valve prosthetic leaflets and cuff FSI analysis to evaluate the paravalvular leak volume BCs: time-dependent pressure waveforms were applied at the ventricular and aortic side, time-dependent flow was imposed at the coronary outlets (dynamic BCs)	The model was neither clinically nor experimentally validated	Regurgitant volume	TAVI valve hemodynamic comparisons between the midway and ventricular positions showed PVL degree of 34.59 ml and 41.61 ml correspondingly, with both magnitudes in the range of severe paravalvular regurgitation. In this case, a post-balloon dilation is recommended to reduce the paravalvular gap
Luraghi *et al* [22]	Average aorta geometryfrom 7 patient specific geometries with severe degree of stenosis	Self—expandable TAV (CoreValve Evolut R). TAV leaflets were included	Aorta: hexahedral elements Native valve: quadrilateral bi-linear shell elements Calcium: tetrahedral elements Stent: hexahedral elements Pericardium leaflets and skirt: quadrilateral bi-linear shell elements	Aorta: anisotropic hyperelastic Native valve: isotropic hyperelastic Calcifications: linear elastic Stent: nitinol Pericardium: linear elastic Blood: Newtonian fluid	TAV procedure FE simulation, six different scenarios of implantation in the same aortic root but different calcifications were modeled. TAV leaflets and cuff were considered in the deployment procedure. Two-way non-boundary fitted FSI simulation performed with ‘operator split’ algorithm Evaluation of the velocity field at three different times: end-diastole, systolic phase and diastolic phase (presence of PVL). Regurgitant volumes were calculated by integrating the curves over the diastolic period. BCs: physiological pressure curves at the ventricular inlet and aortic outlet (dynamic BCs)	The model was neither clinically nor experimentally validated	Blood flow rate curves and regurgitant volumes	The hypothesis that the calcification patterns strongly affect the PVL estimation is confirmed. Calcification patterns appear to influence the proximal deployment of the stent, favoring the appearance of PLV. Radial calcification patterns appear to have a more significant influence with respect to coaptation patterns as the results show
Pasta *et al* [24]	9 pre-implantation BAV CTs	Balloon- expandable TAVs (Edwards SAPIEN 3 and SAPIEN 3 Ultra). Devices were reconstructed by means of micro-CT images. Pericardium leaflets were included	TAV stent: structured- hexahedral solid elements TAV skirt: quadrilateral shell elements Aorta and native valve: quadrilateral shell elements Calcium: hexahedral and tetrahedral solid elements	Aortic root and native leaflets: isotropic hyperelastic (two-terms Yeoh constitutive relation) Calcific plaque: linear elastic Stent: elasto-plastic Pericardial leaflets: linear elastic Pericardial skirt: elasto-plastic Blood: incompressible Newtonian fluid	FE TAV frame deployment simulation, after a pre-TAVI scenario simulation imposing a uniform transmural pressure difference on BAV leaflets. Prosthetic valve leaflets mapping onto deployed stent frame. FSI based on the smoothed particle hemodynamic method to simulate valve leaflet dynamics throughout the entire cardiac cycle PVL degree computation as the flow circulating into the leakage gap area between the aortic wall and the stent frame Simulations validation with post-operative imaging BCs: physiological pressure gradient from literature data (dynamic BCs)	The model was validated against CT and transoesophageal echocardiography images. The predicted values of eccentricity, device expansion, and PVL were in agreement with those clinically	Mean particle velocity, flow circulating into the leakage gap area	Beyond numerical issues, the detailed analysis of PVL has revealed insights on TAVI in stenotic BAV patients that cannot be obtained by conventional imaging. This study represents a further step towards the use of personalized simulations for the virtual planning of TAVI, aiming at improving not only the efficacy of the heart valve implantation but also the exploration of borderline application as the TAVI in bicuspid patients
Dowling *et al* [31]	50 pre-operative CT scans of patients with BAV	Self-expandable TAVs (Evolut R and Evolut PRO). Prosthetic valves reconstruction and leaflets inclusion was not specified	Not specified	Aortic tissue: elastic Native Leaflets: linear elastic Calcifications: elastoplastic	FE TAV deployment simulations at five different sizing and positioning strategies Post-implantation CFD simulations and resulting flow evaluation to identify THV size and implant depth that minimized predictedparavalvular regurgitation. BCs: fixed pressure gradient of 32 mmHg, derived from a population sample (static BCs)	The model was validated against echocardiography. Predicted PVL showed good agreement with clinical observations	Regurgitant flow	Patient-specific THV sizing and positioning were associated with reduced predicted paravalvular regurgitation when compared to different THV sizing and positioning strategies. It supports the finding that optimal patient-specific THV sizing and positioning is associated with improved clinical outcomes in BAV cases
Finotello *et al* [25]	4 BAV pre-implant CTs	Self—expandable TAVs (2 Acurate NEO—2 Evolut R) reconstructed from micro-CT scans of real samples. The pericardium was not included	Aortic root and calcifications: tetrahedral elements Native leaflets: shell elements Stent: hexahedral elements Catheter: quadrilateral surface elements	Aortic root and native leaflets: non-linear isotropic hyperelastic Calcifications: isotropic linear elastic Stent: super-elastic nitinol	FE TAV deployment simulation after a preliminary simulation of native valve opening. A catheter is present in the simulation Evaluation of the paravalvular orifice area by summing the area of the holes between the inner aortic wall (and the calcifications) and the outer surface of the stent	The model was neither clinically nor experimentally validated	Paravalvular orifice area	This study showed that high conformability devices adapted better to the elliptic geometry of the annulus, but higher radial force ones are better in pushing the calcium blocks outwards, providing a better coverage of the annulus, resulting in an overall lower paravalvular orifice area. The authors could suggest that high radial force is a more desired characteristic in a device deployed in BAV patients
Nappi *et al* [27]	4 pre-operative CTs. Two cases were of postoperative device failure	2 Self-expandable TAVs (CoreValve) and 2 balloon-expandable TAVs (Sapien 3). The models were obtained from high-resolution micro-CT images of the actual devices after their expansion. Leaflets were excluded	Aortic root: tetrahedral elements TAV stents: 3D geometries	Native leaflets: isotropic hyperelastic (St. Venant-Kirchhoff) Aortic root: hyperelastic Calcifications: linear elastic Stent (SE): nitinol Stent (BE): elastoplastic with isotropic hardening	FE TAV deployment simulations Comparison between FE simulations and post-operative CTs Calculation of the mismatch between the expanded stent and the internal aortic root surface through effective orifice area	FE simulations were validated against post-operative CT scans. The qualitative comparison indicated good agreement, supporting the accuracy of the numerical model	Effective orifice area	Sites of PVL were in correspondence with regions of geometrical alterations and mainly situated between the non-expanded stent and the internal aortic root. This study permitted to generate hypotheses on the association between post-procedural persistent calcification and the risk of PVL
Pasta *et al* [28]	6 pre-implantation BAV CTs	Self-expandable TAV (Evolut Pro). Device model was reconstructed from CT imaging. TAV leaflets were included	TAV stent: structured- hexahedral solid elements Aorta and native valve: quadrilateral shell elements Calcium: hexahedral and tetrahedral solid elements	Aortic root and native leaflets: hyperelastic with isotropic materials (two-terms Yeoh constitutive relation) Calcific plaque: linear elastic Stent: nitinol alloy Pericardium: linear elastic Blood: incompressible Newtonian fluid	Simulation of pre-TAVI configuration FE TAV deployment simulations FSI analysis using the smoothed particle hemodynamic method for simulating prosthetic valve dynamics and qualitatively assessing region of PVL. BCs: pressure gradient between the left ventricle and aorta generated by representative physiological pressure profiles from literature data (dynamic BCs)	The model was neither clinically nor experimentally validated	Flow velocity maps	A computational framework for the analysis of the structural and hemodynamic performance of THV in patients with severe BAV stenosis is proposed. Flow velocity maps were investigated to visualize the regions at highest risk of PVL. This study represents a further step towards the assessment of the efficacy and safety of TAVI in bicuspid patients
Pasta *et al* [29]	2 pre-implant CTs of BAV patients with severe stenosis	One self-expandable TAV (Evolut PRO) and one balloon-expandable TAV (SAPIEN 3). The stent frames were acquired from micro-CT scans and a general reverse engineering approach allowed to generate the model of each device. Prosthetic valve leaflets were included	Aortic root and native BAV leaflets: quadrilateral shell elements Calcium: combination of hexahedral and tetrahedral elements Stents: hexahedral elements Skirt: quadrilateral shell elements Prosthetic valve leaflets: planar sheets	Stent (BE): elastoplastic with isotropic hardening Stent (SE): pseudo-elastic metallic Skirt: elasto-plastic Aortic root and native BAV leaflets: isotropic hyperelastic (Yeoh 2nd order) Calcifications: linear elastic Prosthetic valve leaflets: isotropic hyperelastic Blood: Newtonian fluid	FE TAV deployment simulation Mapping of the prosthetic valve leaflets onto the implanted stent frames at initial stress-free closed configuration FSI based on the smoothed particle hemodynamic method for estimating the PVL. BCs: Physiological pressure waveforms (dynamic BCs) to obtain the pressure gradient between the left ventricle and the aorta.	The model was neither clinically nor experimentally validated	Flow jet	This study offers a useful computational approach to predict several clinically relevant information of TAVI in BAV patients. The findings suggested that the deployment of the Evolut Pro THV could lead to asymmetric and elliptical deployment, which may increase the risk of PVL compared to the SAPIEN
Anam *et al* [42]	3 pre-operative BAV CTs	Self-expandable TAVs (CoreValve, Evolut R and Evolut Pro+). TAV leaflets were included. Valve design geometry was not specified	TAV stent: hexahedral solid elements Pericardium: quadrilateral shell elements Aortic root: tetrahedral solid elements Native valve: quadrilateral shell elements	Aortic root: hyperelastic Native leaflets and calcific plaque: linear elastic TAV Stent: nitinol alloy TAV leaflets and cuff: linear elastic Blood: two-phase Newtonian fluid	FEA simulations of TAVI procedure with the original devices as well as with a newest generation device (Evolut Pro+) Mapping of prosthetic leaflets CFD studies to assess the PVL degree and location Validation with post-operative echo data BCs: Patient-specific transvalvular pressure gradients obtained from an *in-vitro* flow study (dynamic BCs) using physiological conditions (cardiac output = 5 l min^-1^, heart rate = 60 bpm, mean aortic pressure = 100 mmHg)	In-silico results were validated against post-TAVI two-dimensional echo-doppler data. The in-silico results of PVL severity were consistent with echocardiography data, with a slight overestimation observed in one case	Regurgitant volume	A computational framework to analyze the risk of post-TAVI PVL in BAV patients was developed. Significant decrease in PVL was noticed after implantation of the newest generation device in the same cases. An overall good agreement was found between the clinical and the in-silico data, serving as clinical validation of the models and complementing the clinical data with information that is beyond the reach of clinical modalities
Anam *et al* [43] 2022	3 BAV pre-implant CTs	Three self-expandable TAVs (2 CoreValve—1 Evolut R). Geometric models were reconstructed using an in-house MATLAB code andANSYS Spaceclaim. TAVI leaflets were included	Aorta: shell elements Calcifications and native valve: tetrahedral elements Stent: hexahedral elements Pericardium and skirt: shell elements	Stent: Nitinol alloy Pericardium: linear elastic Aortic tissue and native valve: isotropic hyperelastic Calcifications: linear elastic	FE TAV deployment simulation Mapping of prosthetic leaflets CFD simulations to study the PVL generated during the diastolic flow phase Visualization of the streamlines and calculation of the regurgitation volume to evaluate PVL *In vitro* validation BCs: Pressure at the aortic side of the leaflets was obtained from the *in vitro* flow studies in the patient-specific model (dynamic BCs)	Both the structural and fluid simulations were validated *in vitro* using a patient-specific flexible 3D-printed model. PVL data from patient-specific in silico models were compared with post-TAVR echo-Doppler measurements to validate the computational models. PVL data from the numerical model showed an overall good agreement with echo-Doppler measurements. *In vitro* and in silico flow data exhibited similar trends	Velocity streamlines and regurgitant volume	Development of a workflow that allows patient specific *in silico* and *in vitro* modeling of TAVI procedure and assessment of the interaction between the TAVI device and the aortic root, creating a complementary relationship between these models. *In vitro* and in silico flow data exhibited similar trends in PVL flowrate and the regurgitant volume
Dowling, *et al* [30]	203 pre-operative CT scans including BAV patients	Self-expandable TAVs (CoreValve, Evolut R andEvolutPRO). Prosthetic valves reconstruction and leaflets inclusion was not specified	Not specified	Aortic tissue: elastic Native Leaflets: linear elastic Calcifications: elastoplastic	FE TAV deployment simulations at 0 mm (annular), 4 mm (standard) and 8 mm (deep) implant depth Post-implantation CFD simulations and calculation of the mean predicted PVL from the three simulations Patients’ classification into those in which computer simulation predicted no significant PVL (predicted PVL from CFD <16.0 ml s*^-^*^1^) and those where computer simulation predicted significant PVL (predicted PVL from CFD 16.0 ml s*^-^*^1^) Kaplan-Meier analysis to estimate the association between death from any cause and CFD-predicted significant PVL. BCs: fixed pressure gradient of 32 mmHg, derived from a population sample (static BCs)	The model was validated against transthoracic echocardiography, with predicted paravalvular leak values consistent with clinical findings	Regurgitant flow	Computer simulation may be used to identify patients at risk for the development of moderate PVL after TAVI, and may identify patients at risk for adverse long-term outcomes, such as short-term and long-term mortality. Predicted PVL from CFD was an independent risk factor for death from any cause
Li *et al* [32]	1 pre-implant CT of a male patient diagnosed as severe aortic stenosis	Balloon expandable TAV (Sapien3) and self-expandable TAV with cuff (CoreValve). TAV devices were reconstructed in SolidWorks based on the dimensions of the actual devices. Prosthetic leaflets were included	Stents: hexahedral elements Aorta: wedge grids Calcium and native valve: tetrahedral elements Balloon and pericardium: shell elements Cuff: membrane elements	Stent SE: nitinol alloy Stent BE: elastic-plastic Aorta, calcifications, cuff and balloon: linear elastic Pericardium: isotropic hyperelastic Blood: weakly compressible Newtonian fluid	FETAVdeployment simulation for both types of valves Prosthetic leaflets mapping FSI simulation solved with the immersed boundary method Localization and evaluation of the paravalvular gaps through the blank area between the stent and the aortic root on the annulus plane BCs: at the aortic inlet the left ventricle pressure was applied, while the aortic pressure was applied at the outlet. The curves are obtained from literature (dynamic BCs)	The model was neither clinically nor experimentally validated	Paravalvular gaps	A much smaller postprocedural paravalvular gaps area was found for balloon-expandable valve, which indicated a lower risk and degree of postoperative paravalvular leak for balloon-expandable valve models
Prisco *et al* [33]	One pre-implantation CT	Balloon- expandable TAV(SapienXT).In the ex vivo implantation TAV leaflets were not included. TAV reconstruction design was not specified	Not specified	Blood: incompressible Newtonian fluid Other components: not specified	Anatomical geometry 3D printing. Aortic valve leaflets were excluded TAV ex vivo implantation CT scan and virtual model reconstruction Serial CFD simulations running iteratively, increasing the percent occlusion of the PVL Regurgitant volume calculation by quantifying the flux through a plane drawn halfway through the pathway of the simulated PVL BCs: representative waveforms from the aorta and left ventricle fit to patient data (dynamic BCs)	The model was neither clinically nor experimentally validated	Regurgitant volume	A 3D printed and computational approach able to predict post procedural PVL in TAVI patients was developed. Additionally, it was shown that the diseased native valve leaflet anatomy plays a significant role in reducing PVL in patients undergoing TAVI
Dowling *et al* [35]	16 pre-operative CT scans of BAV patients	Self-expandable TAVs (Evolut R and Evolut PRO). Prosthetic valves reconstruction and leaflets inclusion was not specified	Not specified	Aortic tissue: elastic Native Leaflets: linear elastic Calcifications: elastoplastic Other components: not specified	FE TAV deployment simulations of both an appropriately sized THV and a downsized THV at a high (0 mm) and a medium (4 mm) implant depth CFD simulations to evaluate PVL Patients’ classification into those where computer simulation predicted either none-to-mild or moderate-to-severe PVL Computer simulation output and clinical information revision by the Heart Team to guide patient treatment strategy, THV sizing and positioning. BCs: fixed pressure gradient of 32 mmHg, derived from a population sample (static BCs)	The model was validated against CT, showing agreement in leaflet morphology, raphe location, and calcium distribution	Regurgitant flow	Patient-specific computer simulation may be used to guide the most appropriate treatment modality for patients with BAV. The usage of computer simulation to guide THV sizing and positioning was associated with favorable clinical outcomes
Meng *et al* [39]	6 pre-operative CTA image data, one patient had a BAV	Self-expandable TAVs (Venus A-Valve). The model was reconstructed from CT images. Valve leaflets were not included	Calcium: tetrahedral elements Aortic wall and native valve: triangles Stent: beam elements	Aortic wall, leaflets, and calcifications: linear elastic Venus A-valve: nitinol alloy Blood: Newtonian fluid	FE TAV deployment simulations CFD simulations employing Lattice Boltzmann Method to calculate the regurgitation caused by PVL BCs: average diastolic aortic pressure of 10 000 Pa applied for inlet condition in ascending aorta side and diastolic left ventricular pressure of 2000 Pa applied for outlet in ventricle side (static BCs)	The model was neither clinically nor experimentally validated	Regurgitation flow	The occurrence of paravalvular leak was mainly concentrated at the commissure edge of the native valve, which was consistent with the location of the gap formed by stent inadequate apposition. Using the Venus A valve stent offered greater post-implantation support and reduced perivalvular leakage risk
Spanjaards *et al* [40]	2 synthetic aortic root geometries of an average male and female patient	Self-expandable TAV (CoreValve Evolut). Three different stent sizes were considered. TAV stents were reconstructed based on micro-CT images. TAV leaflets in closed configuration were included	Stent: beam elements All the other components: shell elements	Stent: nitinol alloy Catheter and guiding cylinder: linear elastic Aortic tissue: hyperelastic Neo-Hookean material Blood: incompressible Newtonian fluid Skirt and TAV leaflets: rigid body in CFD simulations	FE TAV deployment simulation. Prosthetic leaflets mapping after deployment Leakage volume reconstruction from leakage slices at different axial heights in the region of interest MAP adaptation according to leakage volume to obtain proper pressure boundary conditions Efficient leakage model validation with *in vitro* experiments and CFD simulations. BCs: pressure gradient adjusted on the leakage slice with the smallest maximum gap size (static BCs)	The model was validated with *in vitro* experiments. The validation results demonstrated good reliability of the model	Regurgitant volume	This paper showed that the efficient leakage model can be used to give a fast indication of the risk of PVL with sufficient accuracy

**Table 4:** Methodological aspect of the conduction abnormalities prediction, focusing on the model discretization, boundary conditions, validation details, and conclusions of the articles [CFD = computational fluid dynamics, FE = finite element, MSCT = multi slice computed tomography, PPM = permanent pacemaker, SE = self-expandable, BE = balloon-expandable, CT = computed tomography, BC = boundary conditions, LVOT = left ventricular outflow tract, TAV = transcatheter aortic valve, BAV = bicuspid aortic valve, THV = transcatheter heart valve, TAVI = transcatheter aortic valve implantation].

Author	Input data	Device model	Model discretization	Material models	Simulation steps and boundary conditions	Model validation	Analyzed parameter	Conclusion
Rocatello *et al* [45]	112 pre-operative CTs of severe aortic patients	Self-expandable TAVs (CoreValve and Evolut R). TAVs frames were reconstructed from optical microscopy measurements and micro-CT images. TAV leaflets were not included	Not specified	Aortic wall and native leaflets: linear elastic Calcifications: stiffer elastic material with perfect plasticity Stent frame: nitinol alloy	FE deployment simulations Extraction of the force exerted on the anatomy Evaluation of maximum contact pressure and contact pressure index (i.e. the percentage of this region of interest subjected to contact pressure) were calculated in the region of interest (i.e. the area between the membrane septum and the plane 15 mm below the annulus) Statistical analysis of correlation between anatomic baseline characteristics, procedural parameters, maximum contact pressure, pressure index and the development of post-operative conduction abnormalities	The model was neither clinically nor experimentally validated	Maximum contact pressure, contact pressure index	Patient-specific computer simulations revealed that maximum contact pressure and contact pressure index are associated with new conduction abnormalities after CoreValve/Evolut R implantation and can predict which patient will have a conduction abnormality after TAVI. Implantation depth, instead, does not seem to represent the driving force of the development of new conduction abnormalities
Dowling *et al* [15]	37 pre-implant CTs of BAV patients	Self-expandable TAVs (5 CoreValve, 16 EvolutR, 2 Evolut PRO) and mechanical-expandable TAVs (14 Lotus). Stents’ morphology was derived from micro-CT scanning Strut width was obtained from optical microscopy or based on data shared by the device manufacturer. TAV leaflets inclusion was not specified	Not specified	Aortic tissue: elastic Native Leaflets: linear elastic Calcifications: elastoplastic TAV: not specified Blood: not specified	FE TAV deployment simulations, including pre- and post-dilatation Extraction of the force exerted on the patient anatomy from the FEA output Measurement of maximum pressure exerted by the THV Statistical analysis	The study validated patient-specific computer simulations comparing predicted contact pressure with periprocedural electrocardiograms to assess major conduction disturbances. The results showed good accuracy of the model	Contact pressure	The results obtained have identified optimal cutoffs for the development of major conduction abnormalities, which were similar to previously reported values derived from work in tricuspid aortic valve morphology
Rocatello *et al* [17]	62 pre-operative MSCTs of patients with severe aortic stenosis	Mechanically expandable Lotus devices. Accurate device models of all Lotus valve sizes were generated based on information provided by the device manufacturer. Device leaflets were not included	Aortic root: triangular elements Native valve and calcific-ations: prism elements	Aortic tissue: elastic Native Leaflets: linear elastic Calcifications: elastoplastic	FE TAV deployment simulations Extraction of the pressure exerted by the device on a selected region of interest of the LVOT (where the atrioventricular conduction system is located) Statistical analysis	In-silico results were compared to post-operative clinical data based on echocardiography and angiography. The results obtained showed good accuracy of the model	Maximum contact pressure and contact pressure index	Combining both maximum contact pressure and contact pressure index enhances the prediction of new conduction abnormalities after Lotus valve implantation
Bosi *et al* [20]	28 pre-implant CTs	Ballon-expandable TAVs (14 SapenXT) and self-expandable TAVs (14 CoreValve revalving System). Both TAVI stent models were designed in their expanded configuration starting from micro-CT scans. Leaflets were not included	Stent (BE and SE): beam elements Balloon: membrane elements Aortic root and native leaflets: shell elements Calcium: tetrahedral elements	Stent (BE): elastoplastic Stent (SE): nitinol Aortic root and native leaflets: linear elastic Calcifications: elastoplastic	FE TAV deployment simulations after pre-dilatation simulations Evaluation of max principal strains from the FE model	The FE model employed in this study had been previously validated [48]	Max Principal Strains	The Max Principal Strains parameter assessed below the coronary ostia both in terms of average and max value, appeared to be a good predictor for onset of conduction abnormalities leading to PPM implantation in this patient population; the parameter was the highest for the patient who underwent PPM, thus providing a potential new monitoring parameter
Dowling *et al* [30]	80 pre-operative CT scans of TAVI patients	Self-expandable TAVs (27 Evolut R and 53 Evolut PRO). Prosthetic valves reconstruction and leaflets inclusion was not specified	Not specified	Aortic tissue: elastic Native leaflets: linear elastic Calcifications: elastoplastic	FE TAV deployment simulations Extraction of the pressure exerted by the THV on the native anatomy and evaluation of the percentage of the left bundle branch subject to pressure (contact pressure index)	Validation was performed by comparing the computer simulation’s predicted conduction disturbance (based on contact pressure indices and maximum pressure on the conduction system) with the patient’s postprocedural ECG findings. The results showed good accuracy of the model	Maximum contact pressure, contact pressure index	Patient-specific computer simulation may be used to identify patients at risk for conduction disturbance after TAVI with current generation self-expanding THVs. This technology could potentially be used to plan and guide procedural aspects to minimize the risk of conduction disturbance and its associated adverse clinical outcomes
Reza *et al* [34]	2 pre-TAVI cardiac CT scans. One patient required a permanent pacemaker implantation	Two balloon-expandable TAVI devices (SAPIEN and SAPIENXT). TAV prosthetic leaflets were not included. Stent geometry design was not specified	Aortic wall, leaflets, and calcium deposits: tetrahedral elements Stents: hexahedral elements Balloon: quadrilat-eral shell elements	Stents: bilinear elasto-plastic Balloon: linear elastic Aortic wall and native leaflets: isotropic hyperelastic Calcification deposits: linear elastic	FE TAV deployment simulations Analysis of mechanical factors such as contact force, contact pressure and strain on the membrane septum, taken in consideration for the risk of conduction abnormalities Comparison between a patient that required permanent pacemaker implantation and a control case that did not require it	The model was neither clinically nor experimentally validated	Area-weighted average maximum principal logarithmic strain, contact force and contact pressure, contact area between the region of interest and the TAVI prosthesis, contact pressure index, implantation depth	This study analyzed anatomical and mechanical factors associated with conduction abnormalities after TAVI. The findings indicate that the anatomical factors play important role in increasing stresses and strains on the conduction fibers. Area-weighted average maximum principal logarithmic strain, contact force and contact pressure index in the membrane septum, were able to correctly assess the post-TAVI conduction abnormalities risk
Dowling *et al* [35]	16 pre-operative CT scans of BAV patients	Self-expandable TAVs (Evolut R and Evolut PRO). Prosthetic valves reconstruction and leaflets inclusion was not specified	Not specified	Aortic tissue: elastic Native Leaflets: linear elastic Calcifications: elastoplastic	FE deployment simulations of both an appropriately sized THV and a downsized THV at a high (0 mm) and a medium (4 mm) implant depth Extraction of the pressure exerted by the THV on the native anatomy and evaluation of the percentage of the left bundle branch subject to pressure (contact pressure index) Patients’ classification into those where computer simulations predicted either none or major conduction disturbance, based on previously validated a cut-off of 14% Computer simulation output and clinical information revision by the Heart Team to guide patient treatment strategy, THV sizing and positioning	The model was validated against CT, showing agreement in leaflet morphology, raphe location, and calcium distribution	Maximum contact pressure, contact pressure index	Patient-specific computer simulation may be used to guide the most appropriate treatment modality for patients with BAV. The usage of computer simulation to guide THV sizing and positioning was associated with favorable clinical outcomes
Meng *et al* [39]	6 pre-operative CT angiography image data, one patient had a BAV	Self-expandable TAVs (Venus A-Valve). The model was reconstructed from CT images. Valve leaflets were not included	Calcium: tetrahedral elements Aortic wall and native valve: triangles Stent: beam elements	Aortic wall, leaflets and calcifications: linear elastic Venus A-Valve: nitinol alloy	FE TAV deployment simulations Extraction of the contact pressure index and the maximum contact pressure	The model was neither clinically nor experimentally validated	Contact pressure index and maximum contact pressure	Localized stresses exerted by the device frame on the membranous septum, which is located between the aortic annulus and the bundle of His, may disrupt cardiac conduction and lead to resultant cardiac conduction abnormalities

**Table 5:** Methodological aspect of the coronary obstruction prediction, focusing on the model discretization, boundary conditions, validation details, and conclusions of the articles [FE = finite element, FSI = fluid structure interaction, SE = self-expandable, BE = balloon-expandable, CT = computed tomography, BC = boundary conditions, TAV = transcatheter aortic valve, CAD = computer-aided design, TAVI = transcatheter aortic valve implantation].

Author	Input data	Device model	Model discretization	Material models	Simulation steps and boundary conditions	Model validation	Analyzed parameter	Conclusion
Capelli *et al* [7]	5 CTs of four patients with four different, stenotic bioprosthetic valves previously implanted via conventional surgery and one patient with incompetentnative aortic valve	Balloon-expandable TAV (Sapien). The model was reconstructed resembling the Edwards Sapien device. TAV leaflets were not included	Aortic root: triangular shell elements Stent: hexahedral elements Balloon: membrane elements	Aortic root: Mooney-Rivlin hyperelastic material model Stent: elasto-plastic material model Balloon: linear elastic material model	FE TAVI deployment simulations. For four patients TAVI stent was deployed in morphologies that included a bioprosthetic valve previously implanted. In the patient with incompetent native aortic valve, the stent was placed in three different positions within the aortic root to test the influence of the landing zone on potential occlusion of the coronary arteries. Residual stresses (400 kPa) were included in the model to take into account the pre-stretching of the aortic root Calculation of the minimum distance of the Sapien device/aortic valve leaflets from the coronary ostia to evaluate the occlusion of the coronary arteries	The model was neither clinically nor experimentally validated	Minimum distance of the Sapien device/aortic valve leaflets from the coronary ostia	With the presented computational approach, an assessment of the risk of coronary occlusion could be quantified. By measuring the distance between device and coronary ostia, in three models comparable values with physiological distances from native aortic valve leaflets (i.e. 12 mm) have been found. The stent implanted in the most distal position is the closest (3.1 mm) to the coronaries. Further fluid dynamic analyses could study in depth the potential interference of the TAVI device to the coronary flow
Kandail *et al* [17]	1 pre-operative CT	Self-expandable TAV (CoreValve). The device model was reconstructed from literature data. TAV leaflets were included	TAV valve: tetrahedral elements TAV stent: hexahedral elements Other components: not specified	Pericardium: linear elastic Stent frame: nitinol alloy Aortic wall: rigid Blood: incompressible Newtonian fluid	FE TAV annular and supra-annular deployment simulations for comparison with subsequent device deformation TAV leaflets mapping FSI simulations in both implantation configurations with a finite-volume based sub-grid geometry resolution method Instantaneous velocity and wall shear stress patterns evaluation at three distinct time points during the cardiac cycle (peak-systole, mid-systole and minimum flow) BCs: time-dependent pressure waveform taken from literature (dynamic BCs)	The model was neither clinically nor experimentally validated	Instantaneous velocity and wall shear stress patterns	CoreValve deployment location has a direct and considerable effect on the manner in which inflow is redirected to the coronary arteries during peak- and mid-systole. As compared to the annularly deployed CoreValve, the supra-annularly deployed CoreValve led to regions of malposition before the aortic root and only the paravalvular flow through these malposed regions was redirected to the coronary arteries during peak- and mid-systole. These results may have important clinical implications given the role of aortic hemodynamic in dilation and the pro-atherogenic nature of wall shear stress alterations in the coronary arteries
Oks *et al* [36]	Aortic root model was adapted from an existing parametric model and a simplified model of the coronary tree was included	Self-expandable TAV (CoreValve). A CAD file was designed, including the prosthesis leaflets	Not specified	TAVI leaflets: hyperelastic (Neo-Hookean) Aortic wall, stent, TAVI cuffs and skirt, coronary arteries, and native valves: rigid Blood: Newtonian incompressible fluid	TAV deployment simulations using morphing functions repeated at three different commissural alignment angles. TAV leaflets included. Two-way immersed boundary FSI simulations BCs: pressure waveforms imposed at the model outlets (dynamic BCs) from models in literature	The model was neither clinically nor experimentally validated	Mean systolic effective orifice area, diastolic leaflet von Mises stresses and coronary flow rate	In order to optimize coronary perfusion, it is necessary to align TAVI commissures with the native ones at least approximately, avoiding the fully misaligned configuration. To control this aspect of device deployment, it is necessary for novel catheter delivery system designs to allow for axial control

**Table 6:** Methodological aspect of the thrombogenic risk prediction, focusing on the model discretization, boundary conditions, validation details, and conclusions of the articles [CFD = computational fluid dynamics, FE = finite element, ALE = Arbitrary Lagrangian–Eulerian, FEA = finite element analysis, FSI = fluid structure interaction, SE = self-expandable, BE = balloon-expandable, CT = computed tomography, BC = boundary conditions, SE = self-expandable, BE = balloon expandable, BAV = bicuspid aortic valve, TAV = transcatheter aortic valve, TAVI = transcatheter aortic valve implantation].

Author	Input Data	Device Model	Model Discretization	Material Models	Simulation Steps and Boundary Conditions	Model Validation	Analysed Parameter	Conclusion
Nappi *et al* [14]	607 Pre-operative CT images of patients with severe aortic valve stenosis. Patients were subdivided into 3 groups: patients without thrombosis, patients with thrombosis and patients with thrombosis and dislodgment	Self-expandable TAVs (CoreValve) and balloon-expandable TAVs (SapienXT). CoreValve model was reconstructed from micro-CTs	Stent: hexahedral solid elements Catheter: quadrilateral surface elements Native Valve and calcifications: tetrahedral solid elements	Leaflet tissues: simplified isotropic hyperelastic (St.Venant-Kirchhoff) Cardiac root tissue and calcifications: hyperelastic material Stent (SE): nitinol alloy	FE TAV procedure simulations Postprocessing of the simulation results and comparison with follow-up data	The model was neither clinically nor experimentally validated	Device apposition and anchoring that was compared to postoperative CT scan	In patients with thrombosis, FEA revealed refractory bulky calcifications after deployment of the self-expanded valve which did not cover the entire circumference of the annulus, resulting in a large paravalvular orifice. The device was not aligned with the aortic root, this lacking complete basal attachment, and showed stent deformation. It was noted that delayed malposition is often accompanied by a reactive inflammatory/ fibrotic process at the level of the migrated cusps leading to calcification and stenosis of the left coronary ostium. Hence, a strong link between solid uncrushed calcifications, delayed dislodgement and late thrombosis was found
Ghosh *et al* [21]	A validated 3D dynamic model of an adult beating heart that includes physiologically realistic structural and electrophysiological properties (Simulia Living Heart Human Model)	Self-expandable TAV (Evolut R). TAV leaflets were included. TAV design geometry was not specified	TAV stent and leaflets: hexahedral solid elements TAV cuff: combination of hexahedral and tetrahedral solid elements Native valve and calcifications: tetrahedral solid elements	Heart tissue: anisotropic hyperelastic (Holzapfel-Ogden) Native leaflets and pericardium: isotropic hyperelastic (Odgen) Calcific plaque: linear elastic TAV Stent: nitinol alloy Blood: Newtonian fluid	FE TAV implantation simulation at three different implantation depths CFD analysis to calculate thrombogenic potential BCs: time-dependent pressure waveforms applied at the ventricular inlet, aortic and coronary outlets (dynamic BCs)	The model was neither clinically nor experimentally validated	Stress accumulation value on each particle representing a platelet	According to the calculated thrombogenic potential, it was concluded that deploying the TAVI device in the midway position was the optimal implantation approach for this patient specific anatomy
Nappi *et al* [23]	98 patient specific CT images, all patients developed symptomatic thrombosis after TAVI	Self-expandable TAVs (82 1st CoreValve, 7 2nd CoreValve, 9 Portico). TAVs model designwas not specified. Prosthetic leaflets were excluded	Not specified	Native leaflets: linear isotropic elastic Aortic root: hyperelastic Calcium: linear elastic Stent (SE): nitinol alloy	FE TAV deployment simulations Model validation with after-stenting CTs Computing of von Mises average stress distribution	FE simulations were validated against post-operative CT scans. The qualitative comparison indicated good agreement, supporting the accuracy of the numerical model	von-Mises stresses	A Finite Element Analysis investigation can anticipate the presence of calcified refractory blocks, the deformation of the prosthetic stent and the development of paravalvular orifice, and it may prevent subclinical and clinical TAVI thrombosis. Using exact geometry from high-resolution CT scans in association with FEA allows detection of persistent bulky calcifications that may contribute to thrombus formation after TAVI procedure
Hatoum *et al* [26]	6 patient specific CT angiographies	Balloon-expandable TAVs (SAPIEN 3). TAVs model was reconstructed from CT angiography images and micro-CT images. TAV leaflets were included	Fluid domain: tetrahedral elements	Blood: Newtonian Other components: rigid domain	Development of a semi-empirical model to predict leaflet thrombosis CFD analyses to validate and improve the predictive model BCs: Flow waveforms, matched to patient-specific cardiac outputs, were imposed at the aortic inlet while a pressure waveform was imposed at the aortic outlet (dynamic BCs)	The model was neither clinically nor experimentally validated	Flux velocity, stasis volume, vorticity flux based on patient-anatomy, wall shear stress	The model shows promising sensitivity and specificity in its predictions. The model emphasizes the significance of normalized circulation in the neo-sinus as a key predictor of thrombus formation
Nappi *et al* [27]	4 pre-operative CTs. Two cases were of postoperative device failure	2 Self-expandable TAVs (CoreValve) and 2 balloon-expandable TAVs (Sapien 3). The models were obtained from high-resolution micro-CT images of the actual devices after their expansion. Leaflets were excluded	Aortic root: tetrahedral elements TAV stents: 3D geometries	Native leaflets: isotropic hyperelastic (St. Venant-Kirchhoff) Aortic root: hyperelastic Calcium: linear elastic Stent (SE): nitinol Stent (BE): elastoplastic with isotropic hardening	FE TAV deployment simulations Comparison between FEA simulations and post-operative CTs Calculation of the eccentricity index as the ratio between the minor and major elliptical axes	FE simulations were validated against post-operative CT scans. The qualitative comparison indicated good agreement, supporting the accuracy of the numerical model	Eccentricity index (frame distortion) and von-Mises stresses	Calcifications cause prosthetic stent deformation and a non-uniform expansion. The non-uniform expansion, due to persistent bulky calcifications, led to different degrees of eccentricity, which were more significant for the CoreValve devices. This study suggested that geometrical alteration might hamper leaflet mobility and trigger hemodynamical abnormalities and flow turbulence, generating a thrombotic nidus
Anam *et al* [43]	3 pre-operative BAV CTs	Self-expandable TAVs (CoreValve, Evolut R and Evolut Pro+). TAV leaflets were included. TAV valve reconstruction was not specified	TAV stent: hexahedral solid elements Pericardium: quadrilateral shell elements Aortic root: tetrahedral solid elements Native valve: quadrilateral shell elements	Aortic root: hyperelastic Native leaflets and calcific plaque: linear elastic TAV Stent: nitinol alloy TAV leaflets and cuff: linear elastic Blood: two-phase Newtonian fluid	FEA simulations of TAVI procedure with the original devices as well as with a newest generation device (Evolut Pro+) CFD studies to assess the risk of thrombogenicity Validation with post-operative clinical data BCs: Patient-specific transvalvular pressure gradients obtained from an *in-vitro* flow study (dynamic BCs) using physiological conditions (cardiac output = 5 l min^-1^, heart rate = 60 bpm, mean aortic pressure = 100 mmHg)	Both the structural and fluid simulations were validated *in vitro* using a patient-specific flexible 3D-printed model. PVL data from patient-specific in silico models were compared with post-TAVR echo-Doppler measurements to validate the computational models. The in-silico results of PVL severity were consistent with echocardiography data, with a slight overestimation observed in one case	Stress accumulation value on each particle representing a platelet	A computational framework to analyse the risk of post-TAVI flow-induced thrombogenicity in BAV patients was developed. A decrease of the thrombogenic risk was observed with the newest generation device
Oks *et al* [37]	1 Patient-specific CT model on which sinotubular junction size was parametrically adjusted taking sizes from literature	Self-expandable TAV (Evolut). Prosthesis leaflets were included in the FSI simulations only. TAV valve reconstruction was not specified	Stent: hexahedral elements Aortic root, native valve and calcifications: tetrahedral elements Prosthetic leaflets: hybrid linear hexahedron pentahedron solid elements	Stent: nitinol Alloy Aortic wall and leaflets: isotropic hyperelastic (3rd order Odgen) TAV leaflets: hyperelastic (Neo-Hookean) Blood: incompressible fluid	FE TAV deployment simulation, the Neosinus cavity formed post deployment was also modeled TAV leaflets mapping Immersed two-way FSI coupling method Lagrangian platelets tracking to assess the risk of platelet activation BCs: Dirichlet boundary conditions were set at the inlet, while zero traction Neumann boundary conditions were imposed at the outlet (dynamic BCs)	The model was neither clinically nor experimentally validated	Transvalvular pressure gradient, geometric orifice area, stress accumulation on the platelets, Wall shear stress	Smaller sinotubular junction sizes lead to significant under-expansion of the stent, reducing the cross-sectional area at the crown region and impairing hemodynamic performance. This is characterized by decreased geometric orifice area, elevated transvalvular pressure gradient and increased wall shear stress in the ascending aorta, which could contribute to endothelial damage and complications. Larger sinotubular junction sizes, instead, allowed for better stent expansion, improving geometric orifice area and reducing jet velocity and wall shear stress, but also increased platelet residence time and flow stagnation, elevating the risk of thrombosis and leaflet degeneration due to stress accumulation on platelets)
Baylous *et al* [38]	6 pre-TAVI cardiac CT scans of type I BAV patients who underwent TAVI and 3 idealized models	Self-expandable TAVs (Core Valve, Evolut R) and balloon-expandable TAVs (SAPIEN 3). TAV leaflets were included	TAV leaflets: shell elements Aortic root components and stents: tetrahedral elements	Aortic wall and leaflets: hyperelastic (3rd order Odgen) Calcium: linear elastic Blood: Newtonian fluid. TAV leaflets: hyperelastic (Mooney-Rivlin and Ogden, to model xSIBS elastomeric- thermoplastic behavior and pericardial tissue properties) Stents: assumed rigid for the FSI analysis, not specified for the FEA	FE TAVIdevice deployment simulations, including prosthetic leaflets FEM-ALE FSI strong coupling simulations FSI validation using hydrodynamic testing and published data Thrombogenic risk assessment with Lagrangian particle tracking approach and stress accumulation analysis Compare thrombogenic footprints across TAVI idealized designs and patient-specific scenarios BCs: A physiological pressure gradient was applied at the inlet (dynamic BCs), with zero pressure at the outlet	The FSI workflow was validated using derived flow rates, high-speed imaging snapshots, and published data. The in-silico results were consistent with the literature data	Stress accumulation	The study evaluates heart valve dynamics and thrombogenic risk, highlighting a strong link between increased leak flow and thrombogenic risk. Key clinical parameters, such as effective orifice area, leaflet flexion, stresses, flow velocities, and cardiac output, are crucial for assessing and mitigating thrombogenic risk. The findings provide valuable quantitative data to guide procedural planning and allow clinicians to tailor interventions based on patient-specific characteristics

**Table 7:** Methodological aspect of the stent migration prediction, focusing on the model discretization, boundary conditions, validation details, and conclusions of the articles [FE = finite element, FSI = fluid structure interaction, SE = self-expandable, BE = balloon-expandable, CT = computed tomography, BC = boundary conditions, THV = transcatheter heart valve, TAV = transcatheter aortic valve].

Author	Input data	Device model	Model discretization	Material models	Simulation steps and boundary conditions	Model Validation	Analysed Parameter	Conclusion
Bianchi *et al* [10]	1 pre-operative CT of a retrospective case of valve migration	Balloon-expandable TAV (SAPIEN) created by employing parametric equations that enabled the estimation of the expanded stent configuration. The bioprosthetic leaflets were not included in the deployment models after a sensitivity analysis	TAV stent: brick elements TAV leaflets: triangular shell elements Balloon: quadrilateral shell elements Aorta, native leaflets and calcifications: tetrahedral solid elements	TAV stent: elasto-plastic Pericardium: hyperelastic material model (Odgen, 3rd order) Balloon: linear elastic Aorta and native leaflets: hyperelastic (3rd order Odgen) Calcifications: linear elastic	FETAVcrimping simulations with a comparative analysis of the model to investigate whether the prosthetic leaflets were necessary for accurate modeling of the stent crimping FE TAV deployment simulations at three different locations, excluding the bioprosthetic leaflets from the device model Calculation of the contact forces and area between the stent to the native aortic valve throughout the deployment and recoil phases to assess migration risk	The model was neither clinically nor experimentally validated	Contact area and pressure between the native leaflets and the stent during the deployment and recoil phases	The comparison of the three positions showed that a proximal deployment could lead to valve migration into the left ventricle. This case resulted in lower contact area, which led to higher localized contact pressure and higher stress levels in the native tissue that are likely to dislodge the valve. The distal and midway configurations had comparable outcomes. After further validation, the proposed approach might be used as a predictive tool for procedural planning in order to ultimately prevent prosthesis migration
Wu *et al* [18]	Aorta geometry reconstructed based on the statistics regarding adult aorta sizes obtained from 2D echocardiographic images	Self-expandable TAV (CoreValve). The THV frame is parametrically modeled and validate. TAV leaflets were included	Aorta: solid elements THV leaflets and skirt: shell elements Frame: beam elements	THV Leaflets: anisotropic hyperelastic Frame: elastic (St.Venant-Kirchhoff) Aorta: hyperelastic Blood flow: incompressible Newtonian	FE TAV deployment simulation, including prosthetic leaflets Immersegeometric FSI Extraction of the total radial and static friction forces BC: A physiologically realistic left ventricular pressure profile is applied at the inlet (dynamic BCs)	The model was neither clinically nor experimentally validated	Radial force and friction force	During the FSI simulation, radial and friction forces are computed and studied simultaneously to obtain the ratio of friction force to radial force, which is important for indicating the anchoring capability of the THV. By comparing the computed values to the given coefficient of friction, changes may be made to the THV geometry to lower the risk of migration
Ghosh *et al* [19]	A validated 3D dynamic model of an adult beating heart that includes physiologically realistic structural and electrophysiological properties (Simulia Living Heart Human Model)	Self-expandable TAV (Evolut R). TAV leaflets were included. TAV valve reconstruction was not specified	TAV stent and TAV leaflets: hexahedral solid elements TAV cuff: combination of hexahedral and tetrahedral solid elements Native valve and calcifications: tetrahedral solid elements	Heart tissue: anisotropic hyperelastic (Holzapfel-Ogden) Native leaflets and pericardium: isotropic hyperelastic (Ogden) Calcific plaque: linear elastic TAV Stent: nitinol alloy	FE TAV implantation simulation into the living heart human model at three different implantation depths to evaluate stent anchorage	The model was neither clinically nor experimentally validated	Anchorage contact area and force between the stent frame and the native calcific aortic valve over time	Under the assumption that the larger anchorage area will ensure better anchorage of the TAVI stent with less likely migration, it can be concluded that the stent implanted in an aft position more toward the ventricular side was the optimal implantation depth for the specific anatomic geometry

**Table 8:** Methodological aspect of aortic root rupture prediction, focusing on the model discretization, boundary conditions, validation details, and conclusions of the articles [FE = finite element, CT = computed tomography, TAVI = transcatheter aortic valve implantation, TAV = transcatheter aortic valve].

Author	Input data	Device model	Model discretization	Material models	Simulation steps and boundary conditions	Model Validation	Analysed Parameter	Conclusion
Wang *et al* [9]	Pre-operative CT scans of an actual TAVI aortic rupture case and two high-risk patients	Balloon-expandable TAVs (SAPIEN). The models were generated using depictions in the literature. TAV leaflets were not included in the model	Balloon: membrane elements Other components: hexahedraI, pentahedral, and tetrahedral solid elements	Heart tissue: anisotropic hyperelastic (Holzapfel-Gasser-Ogden model) Calcium: linear elastic TAV stent: elasto-plastic Ballon: linear elastic	FE TAV deployment simulations Analysis of the contact force between the stent and aortic root and deformed geometry of the aortic root analysis	The model was neither clinically nor experimentally validated	Contact force between the stent and aortic root, deformed geometry of the aortic root	From simulation results, it can be seen that pressure and force values were not correlated with the aortic rupture. Thus, aortic rupture mechanism could be different among patients. TAV sizing should be evaluated with patient-specific anatomic features and calcification configurations, which underscored the importance of case-by-case patient-specific analysis of complicated, rare clinical TAVI cases of aortic rupture

## Data Availability

All data that support the findings of this study are included within the article (and any supplementary files).
